# Glycan-binding F-box protein from *Arabidopsis thaliana* protects plants from *Pseudomonas syringae* infection

**DOI:** 10.1186/s12870-016-0905-2

**Published:** 2016-10-04

**Authors:** Karolina Stefanowicz, Nausicaä Lannoo, Yafei Zhao, Lore Eggermont, Jonas Van Hove, Bassam Al Atalah, Els J. M. Van Damme

**Affiliations:** Department of Molecular Biotechnology, Ghent University, Coupure Links 653, B-9000 Ghent, Belgium

**Keywords:** F-box-Nictaba, Glycan, Lectin, *Pseudomonas*, Salicylic acid, Stress

## Abstract

**Background:**

A small group of F-box proteins consisting of a conserved F-box domain linked to a domain homologous to the glycan-binding protein has been identified within the genome of *Arabidopsis thaliana*. Previously, the so-called F-box-Nictaba protein, encoded by the gene *At2g02360*, was shown to be a functional lectin which binds *N-*acetyllactosamine structures. Here, we present a detailed qRT-PCR expression analysis of *F-box-Nictaba* in Arabidopsis plants upon different stresses and hormone treatments.

**Results:**

Expression of the *F-box-Nictaba* gene was enhanced after plant treatment with salicylic acid and after plant infection with the virulent *Pseudomonas syringae* pv. *tomato* strain DC3000 (*Pst* DC3000). β-glucuronidase histochemical staining of transgenic Arabidopsis plants displayed preferential activity of the *At2g02360* promoter in trichomes present on young rosette leaves. qRT-PCR analyses confirmed high expression of *F-box-Nictaba* in leaf trichomes. *A. thaliana* plants overexpressing the gene showed less disease symptoms after *Pst* DC3000 infection with reduced bacterial colonization compared to infected wild type and F-box-Nictaba knock-out plants.

**Conclusions:**

Our data show that the Arabidopsis *F-box-Nictaba* gene is a stress-inducible gene responsive to SA, bacterial infection and heat stress, and is involved in salicylic acid related plant defense responses. This knowledge enriched our understanding of the physiological importance of F-box-Nictaba, and can be used to create plants with better performance in changing environmental conditions.

**Electronic supplementary material:**

The online version of this article (doi:10.1186/s12870-016-0905-2) contains supplementary material, which is available to authorized users.

## Background

F-box proteins represent one of the largest and most diverse protein families in plants, as most species have several hundred representatives [1]. They are named after their highly conserved N-terminal protein-protein interaction motif of approximately 50 amino acid residues, known as the F-box domain. The majority of the F-box proteins function as part of Skp1-Cullin1-F-box (SCF)-type ubiquitin E3 ligases [2] in which the F-box protein comprises the substrate-binding module. F-box proteins are assembled into active SCF complexes through a direct binding of the F-box motif with the SCF core S-phase kinase-related protein 1 (Skp1). Through their variable C-terminal substrate-binding domain, F-box proteins specifically bind to and deliver appropriate substrates to the SCF complex for ubiquitin-mediated proteolysis by the ubiquitin-proteasome system [3].

The impressive number of F-box proteins in plants and the extensive diversity of their C-terminal target-binding domains contribute to the ability of SCF complexes to target a wide variety of substrates. Therefore, it is not surprising that F-box proteins are involved in numerous cellular processes within plant development and stress signaling [4]. Genetic approaches already revealed an essential role for F-box proteins in plant hormone perception and signaling [5]. F-box proteins were also reported to be involved in circadian clock control, photomorphogenesis and flowering [6, 7], leaf senescence [8], self-incompatibility [9] and responses to various (a)biotic stresses [10–12].

Multiple newly discovered F-box proteins (the so-called FBXO proteins) combine various recognition mechanisms which enable tight regulation of substrate selection by the F-box protein and each complementary SCF complex [3]. Amongst these new FBXO proteins, glycan-binding F-box (or FBG) proteins were first discovered in mammals [13], including FBG1, FBG2 and FBG5 which bind to high-mannose *N*-glycosylated proteins [14]. The C-terminal substrate-binding domain of both FBG1 and FBG2 specifically interacts with the inner N,N’-diacetylchitobiose (GlcNAc_2_) core of high-mannose *N-*glycans present on incompletely folded or denatured glycoproteins. Since FBG1 and FBG2 do not target free mannose structures or non-glycosylated proteins, it was concluded that both FBG proteins function as glycan-binding F-box proteins involved in the endoplasmic reticulum associated degradation pathway [15]. In this pathway, proteins which fail to fold correctly or assemble into oligomeric complexes in the lumen of the endoplasmic reticulum are retro-translocated to the cytosol, where they are captured by an SCF^FBG1/2^ complex before degradation by the ubiquitin-proteasome system [16].

Recently, FBG-like proteins were also reported in plants. The *A. thaliana* genome contains multiple genes encoding F-box proteins with a putative glycan-binding or lectin-like domain [17, 18]. These proteins are referred to as the F-box-Nictaba family, since their C-terminal domain highly resembles Nictaba, an *N*-glycan-binding jasmonate-inducible lectin from tobacco [19]. This F-box protein family in *A. thaliana* groups approximately 30 members that share over 90 and 40–64 % sequence similarity in the F-box domain and the Nictaba domain, respectively [4]. Due to the presence of the F-box domain linked to a lectin-like C-terminal substrate-binding domain, it is tempting to speculate that these F-box-Nictaba proteins function as substrate adaptors in an endoplasmic reticulum associated degradation pathway in plants similar to the mammalian FBG proteins. Furthermore, both Nictaba and FBG1 show comparable glycan-binding properties towards the inner core structure of *N*-glycans [14, 20]. However, detailed analysis of the sugar specificities for the *A. thaliana* F-box-Nictaba protein (encoded by the gene *At2g02360*) revealed binding of its Nictaba domain to *N-* and *O-*glycans containing (poly)*N-*acetyllactosamine (Galβ1-3GlcNAc and Galβ1-4GlcNAc) structures, Lewis a, Lewis x and Lewis y motifs [18]. At present, it is not clear if and how the (nucleocytosolic) F-box-Nictaba protein can bind to these structures *in planta. N-*glycan modification with Lewis a motifs takes place in the Golgi apparatus and plant-specific Lewis epitopes were only shown on some extracellular glycoproteins and on membrane-bound glycoproteins present at the plant cell surface [21–23]. Moreover, in contrast to most plant families, the occurrence of Lewis a motifs in *A. thaliana* was reported to be generally low and tissue specific with highest levels in pedicels, stems and nodes and with moderate levels in siliques and the shoot apex. Lewis a structures are not detectable in the leaves of *A. thaliana* plants [24].

In this study, we show that the transcripts for the *A. thaliana* F-box-Nictaba protein encoded by *At2g02360* are up-regulated after plant treatment with salicylic acid (SA), heat stress and upon infection with *Pseudomonas syringae* pv. *tomato* strain DC3000, with preferential activity of its promoter sequence in leaf trichomes. Stress experiments on transgenic *A. thaliana* plants with altered F-box-Nictaba expression suggested a role for *F-box-Nictaba* in plant defense-related signaling pathways.

## Results

### *F-box-Nictaba* gene expression is up-regulated after SA application and heat shock treatment

**Fig. 1:**
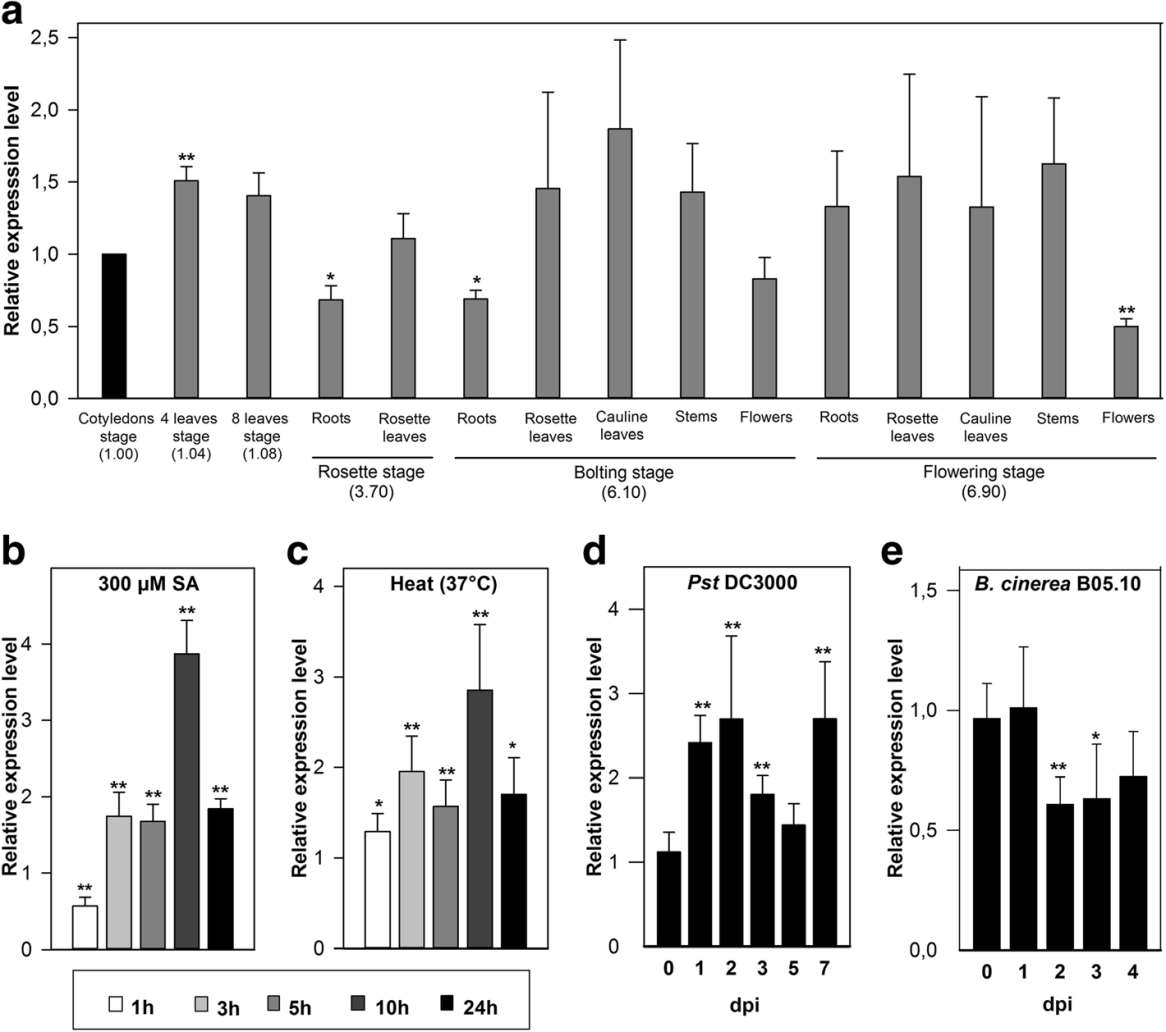
Relative transcript levels of *At2g02360* in WT *A. thaliana* Col-0 plants determined by qRT-PCR analyses of two independent biological experiments, each with multiple technical replicates as pointed out in Methods section; error bars ± SE. Asterisks indicate statistically significant differential expression compared to control samples (*p < 0.05; **p < 0.01). **a**, *At2g02360* expression levels measured in *A. thaliana* plants grown under standard conditions. **b**-**c**, *At2g02360* expression levels in 16-day-old *A. thaliana* seedlings after SA treatment (**b**) or after heat shock (**c**). **d**-**e**, Relative expression of *At2g02360* after infection of 5-week-old *A. thaliana* plants with *Pst* DC3000 (**d**) or with *B. cinerea* strain B05.10 (**e**). Expression levels are compared (**a**) to the *At2g02360* expression level in the cotyledons stage or (b-e) to gene expression levels in the mock-treated plants

The expression pattern of *F-box-Nictaba* was first analysed in wild-type (WT) *A. thaliana* plants grown under laboratory growth conditions. qRT-PCR analyses demonstrated that *At2g02360* was expressed during all developmental stages and in every tissue tested (Fig. 1a). A slightly lower expression of *At2g02360* was observed in roots from young plants and in flowers compared to the youngest stage tested (i.e., cotyledons stage). *In silico* expression analysis indicated that *At2g02360* is a stress-responsive gene (Additional file 1: Figures S1-S2, Table S1 and Text S1). Indeed, the uniform expression profile of *At2g02360* considerably changed when *A. thaliana* plants were subjected to treatments with various plant hormones and several abiotic stress factors (Fig. 1b-c) (Additional file 1: Figure S4). After 1 h (h) of SA treatment, *At2g02360* expression was slightly down-regulated compared to the mock treatment, but *At2g02360* mRNA levels were significantly higher after 3 h with a maximal increase of almost 4-fold after 10 h of SA application (Fig. 1b). Transcript levels in treated plants slightly dropped after 24 h but were still two-fold higher than in the untreated plants. Transcript levels for the SA-inducible transcription factor WRKY70 were up-regulated faster and reached much higher levels compared to *F-box-Nictaba* expression levels (Additional file 1: Figure S3a).

Also heat stress enhanced *F-box-Nictaba* gene expression with a maximal up-regulation of almost 3-fold after 10 h of heat stress compared to control plants (Fig. 1c). However, compared to the *At2g02360* gene the expression of the *Hsp70b* gene, encoding a heat shock-responsive chaperone protein, was enhanced much faster and reached much higher mRNA levels (Additional file 1: Figure S3b). Other hormones and abiotic stress treatments either slightly down-regulated or did not affect *F-box-Nictaba* gene expression at all (Additional file 1: Figure S4).

### *F-box-Nictaba* expression is up-regulated in WT *A. thaliana* plants after *Pseudomonas* infection but is slightly down-regulated by fungal infection

Five-week-old WT *A. thaliana* Col-0 plants were infected either with the virulent hemibiotrophic bacterium *P. syringae* pv. *tomato* strain DC3000 (*Pst* DC3000) or the necrotrophic fungus *Botrytis cinerea* strain B05.10. *Pst* DC3000 infection strongly enhanced the expression of both control genes *WRKY70* and *PR1* in rosette leaves (Additional file 1: Figure S3c). *F-box-Nictaba* mRNA levels were significantly up-regulated approximately 2.5-fold at 1, 2 and 7 dpi compared to mock-sprayed plants. *F-box-Nictaba* expression dropped at 3 and 5 dpi but was still higher than in the mock-treated plants (Fig. 1d). In contrast, *B. cinerea* infection mildly affected *F-box-Nictaba* mRNA levels compared to mock-treated plants (Fig. 1e). At 2 and 3 dpi, *At2g02360* expression levels were reduced almost two-fold. The expression of the control gene *PR1* was not altered significantly (Additional file 1: Figure S3d). In contrast, the expression of the control gene *PDF1.2* was highly up-regulated reaching a 50-fold increase in infected tissues at 3 dpi (Additional file 1: Figure S3d).

### *F-box-Nictaba* promoter is particularly active in leaf trichomes

**Fig. 2:**
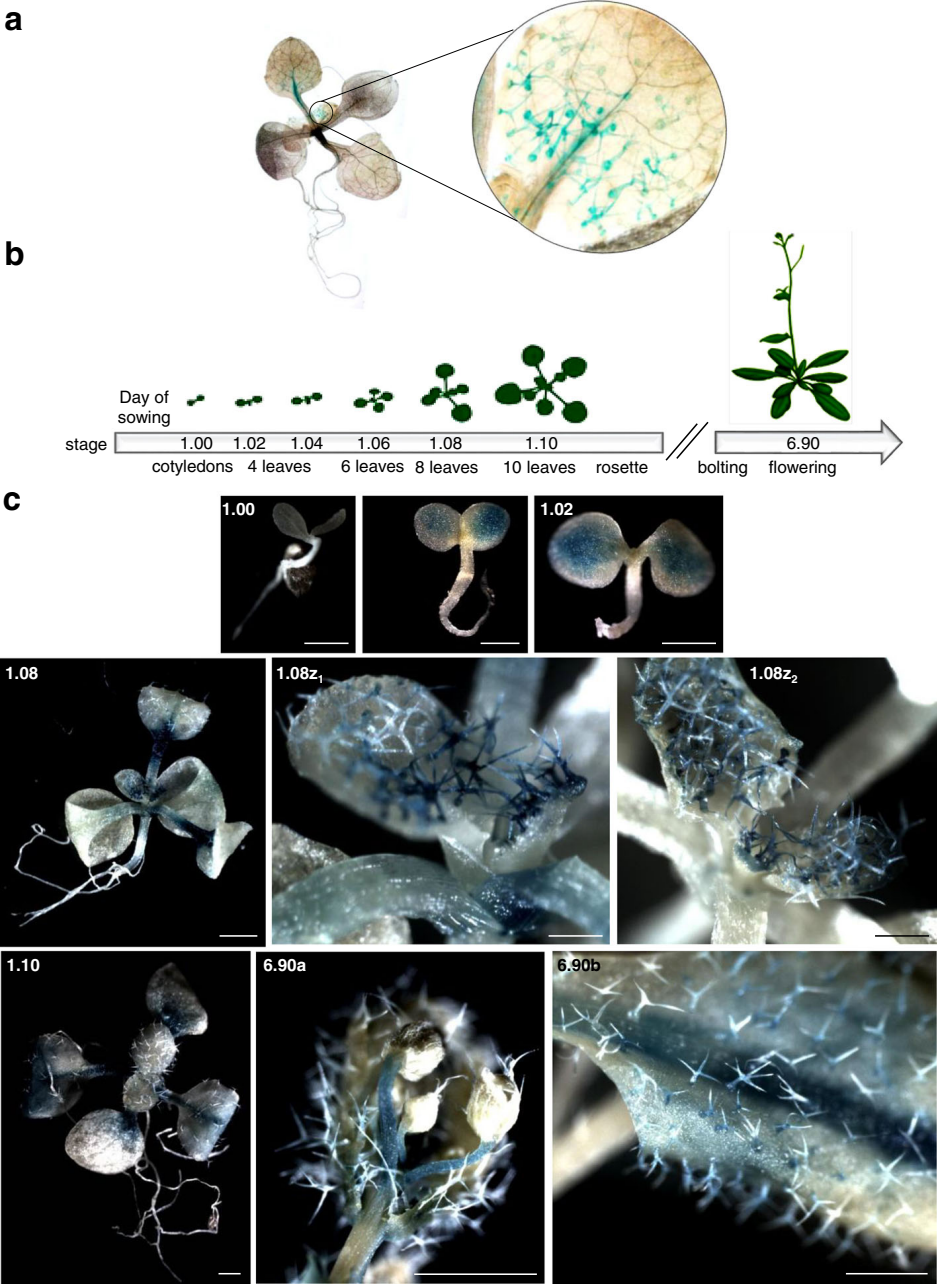
Histochemical analysis of *pAt2g02360:GUS A. thaliana* lines. **a**, GUS staining data for 14-day-old plantlets. **b**, Different developmental plant stages analyzed [46]. **c**, GUS staining data for selected developmental stages with a closer look at trichomes present on new leaves (panel 1.08z) and on flower buds (panel 6.90a); panel 6.90b represents a close-up of rosette leaves. Scale bars represent 1 mm

To test the activity of the 5′-upstream region of the *At2g02360* gene, a promoter *At2g02360*:GUS reporter construct was introduced into *A. thaliana* Col-0 plants and used for histochemical assays. First data revealed a distinct and localized GUS staining in the leaf trichomes of 14-day-old *pAt2g02360:GUS A. thaliana* seedlings (Fig. 2a). Subsequently, plants grown under standard conditions were also analyzed at different developmental stages (Fig. 2b). Overall, plants of different transgenic lines showed comparable GUS staining patterns, but the intensity of the GUS staining differed amongst plants. In the very young seedlings (stage 0.7) no GUS staining was detected (Fig. 2c). In the cotyledons and 2-leaves stage (stages 1.00 and 1.02), plantlets showed a weak GUS activity spread over mesophyll cells. When plants developed (from stage 1.08 onwards), a very intense GUS activity was observed all over the petioles and in the majority of the trichomes present on the shoot meristem and first leaves. GUS staining in the trichomes present on newly developing leaves remained throughout further development of the plants. In plantlets of stage 1.10 and older (i.e., rosette stage), GUS staining was prominently visible in trichomes present on new leaves and in the petioles, the major leaf vein and some parts with mesophyll cells of older leaves. Trichomes present on older leaves also showed GUS staining, especially in those located in close proximity of the petiole, but their GUS staining was less intense compared to the staining detected in trichomes residing on young leaves. In flowering plants (stage 6.90), GUS staining was mainly visible in the flowers, some siliques and in some major veins and trichomes located in close proximity of these veins on rosette and cauline leaves. The CaMV35S:GUS control plants showed an intense blue, homogeneous GUS staining pattern in all organs and tissues of the plants throughout development (Results not shown).

### *F-box-Nictaba* is predominantly expressed in the trichomes of *A. thaliana* plants

**Fig. 3:**
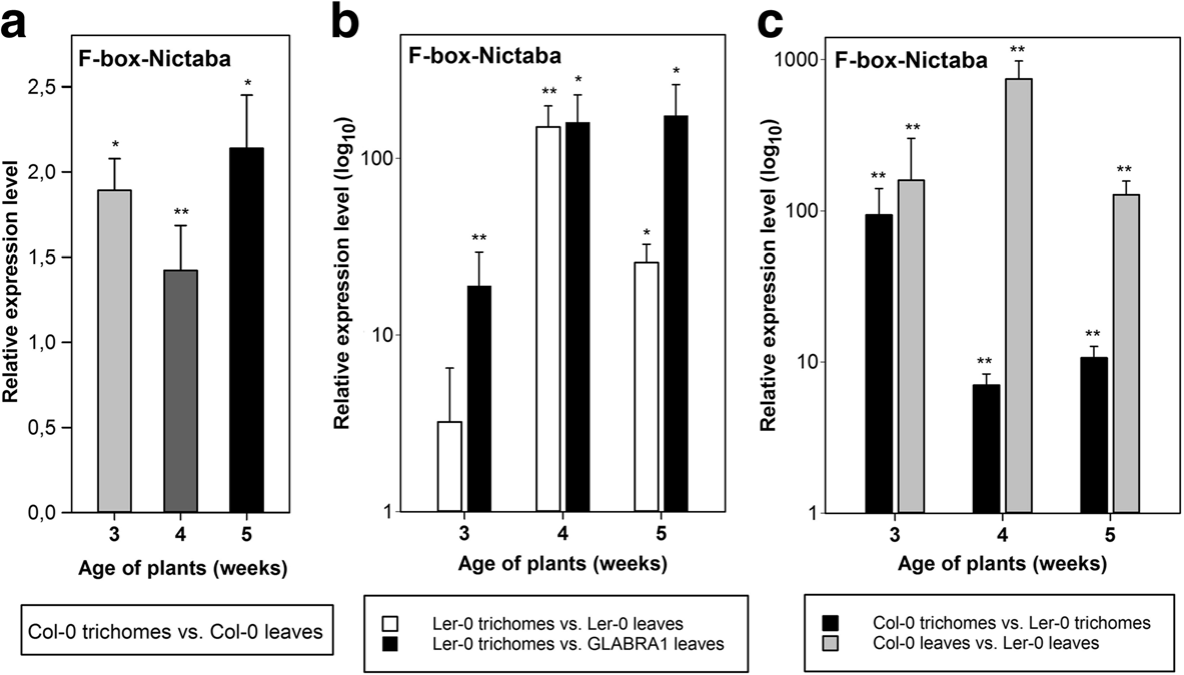
Transcript levels of *At2g02360* in the trichomes isolated from rosette leaves of WT *A. thaliana* plants of Col-0 or Ler-0 ecotype compared to corresponding gene expression levels in complete rosette leaves from WT *A. thaliana* plants of Col-0 or Ler-0 ecotype or of *GLABRA1 A. thaliana* plants. Relative transcript levels were determined in 3-, 4- and 5-week-old plants by qRT-PCR analyses of two independent biological experiments, each with two technical replicates; error bars ± SE. Asterisks indicate statistically significant differential expression compared to control samples (complete rosette leaf material). **a**, gene expression levels in the trichomes of WT *A. thaliana* Col-0 plants compared to complete rosette leaves from WT *A. thaliana* Col-0 plants. **b**, gene expression levels in the trichomes of WT *A. thaliana* Ler-0 plants compared to complete rosette leaves of WT *A. thaliana* Ler-0 plants or compared to complete rosette leaves of *GLABRA1 A. thaliana* plants as well as in complete rosette leaves of WT *A. thaliana* Ler-0 plants compared to complete rosette leaves of *GLABRA1* plants. **c**, Comparison of gene expression levels for *F-box-Nictaba* in the trichomes and in complete rosette leaves of WT *A. thaliana* Col-0 compared to WT *A. thaliana* Ler-0 plants

Given the apparent activity of the *pAt2g02360:GUS* reporter construct in leaf trichomes and the occurrence of putative *cis-*regulatory elements reported to be responsible for trichome-specific gene regulation in the *F-box-Nictaba* promoter sequence including eight MYB-like recognition sites and five T/G-box elements (Additional file 1: Figure S5), the expression of the *F-box-Nictaba* gene was quantified by qRT-PCR in trichomes purified from WT *A. thaliana* Col-0 rosette leaves. *At2g02360* mRNA levels measured in trichomes were slightly higher compared to those measured in unprocessed rosette leaves (containing trichomes) with increased expression in trichomes up to approximately 1.5-fold in 4-week-old plants and 2-fold in 3- and 5-week-old plants (Fig. 3a).

*F-box-Nictaba* gene expression was also analyzed in rosette leaves of mutant *A. thaliana* plants completely lacking trichomes. These *GLABRA1* plants are impaired in the *GL1* gene (*At3g27920*) which encodes a MYB-like transcription factor required for trichome development [25]. Since the *GLABRA1* mutants are made in a Landsberg erecta-0 (Ler-0) background, *F-box-Nictaba* expression was also examined in WT *A. thaliana* Ler-0 plants. Trichome purification from rosette leaves of *A. thaliana* Ler-0 plants yielded similar amounts of trichomes as for *A. thaliana* Col-0 plants. However, the relative expression of the *F-box-Nictaba* gene was much more pronounced in trichomes of *A. thaliana* Ler-0 plants with an up-regulation of 3.2-fold (although not statistically significant), 150-fold and 25-fold compared to total leaf tissue from 3-, 4- and 5-week-old *A. thaliana* Ler-0 plants, respectively (Fig. 3b, white bars). In comparison to *GLABRA1 A. thaliana* plants, *At2g02360* transcript levels were 20-, 160- and 175-fold higher in trichomes from *A. thaliana* Ler-0 plants when compared to total leaf tissue from 3-, 4- and 5-week-old GLABRA1 *A. thaliana* plants, respectively (Fig. 3b, black bars).

The transcript levels for the *F-box-Nictaba* gene were higher in WT *A. thaliana* leaves of plants with a Col-0 background compared to leaves from WT plants with a Ler-0 background, at all developmental stages investigated, irrespective whether the purified trichomes or complete rosette leaves of both ecotypes were assessed (Fig. 3c). The differential expression was most striking when unprocessed rosette leaves were compared (gray bars): *At2g02360* mRNA levels were approx. 160, 740 and 130 times higher in leaves of 3-, 4- and 5-week-old plants of Col-0 ecotype, respectively, compared to leaves of the same age from plants with a Ler-0 background. In the trichomes isolated from 3-, 4- and 5-week-old WT *A. thaliana* Col-0 plants *F-box-Nictaba* gene expression was 90-, 7- and 10-fold higher than in the trichomes of 3-, 4- and 5-week-old plants from WT *A. thaliana* Ler-0, respectively (Fig. 3c, black bars).

### *GALT1* and *FUT13* genes are up-regulated in the trichomes of WT *A. thaliana* Ler-0 plants

**Fig. 4:**
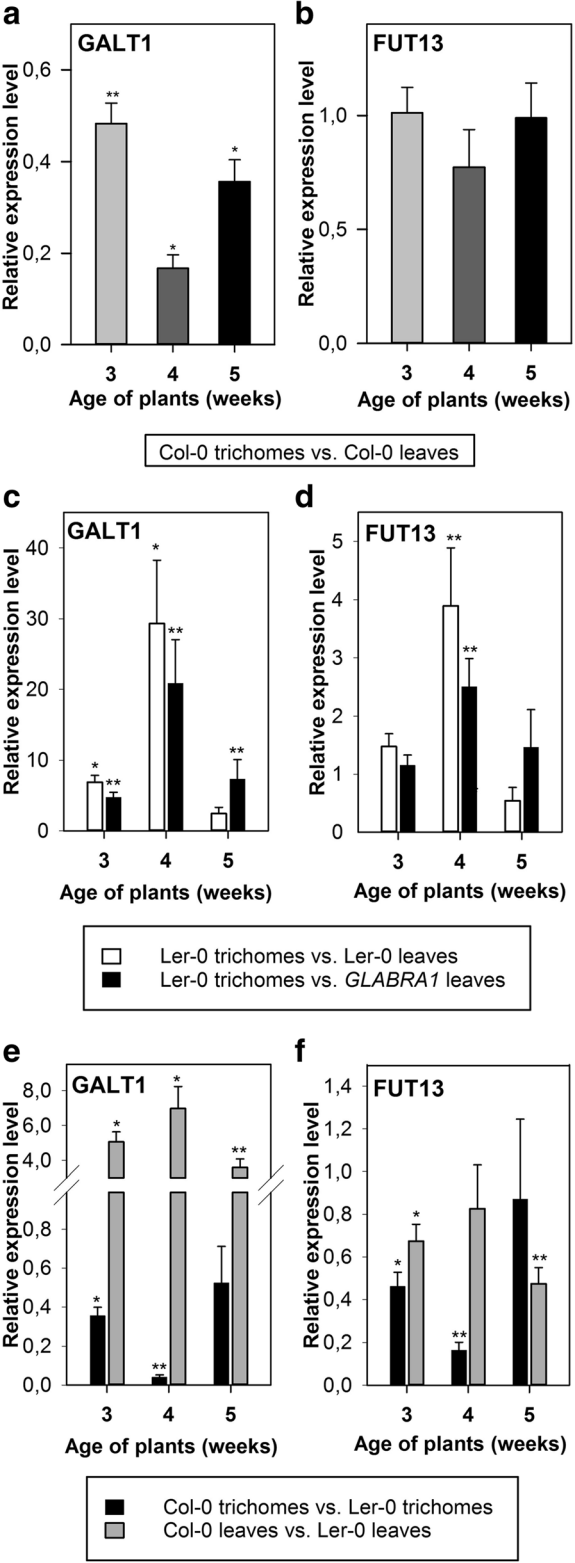
Transcript levels of *GALT1* (*At1g26810*) and *FUT13* (*At1g71990*) in trichomes isolated from rosette leaves of WT *A. thaliana* plants of Col-0 or Ler-0 ecotype compared to corresponding gene expression levels in complete rosette leaves of WT *A. thaliana* plants of Col-0 or Ler-0 ecotype or of *GLABRA1 A. thaliana* plants. Transcript levels were determined in 3-, 4- and 5-week-old plants by qRT-PCR analyses of two independent biological experiments, each with two technical replicates; error bars ± SE. Asterisks indicate statistically significant differential expression compared to control samples (complete rosette leaf material). **a**-**b**, Relative gene expression levels of *GALT1* (**a**) and *FUT13* (**b**) in the trichomes of WT *A. thaliana* Col-0 plants compared to complete rosette leaves from WT *A. thaliana* Col-0 plants. **c**-**d**, Relative gene expression levels of *GALT1* (**c**) and *FUT13* (**d**) in the trichomes of WT *A. thaliana* Ler-0 plants compared to complete rosette leaves of WT *A. thaliana* Ler-0 plants or compared to complete rosette leaves of *GLABRA1 A. thaliana* plants as well as in complete rosette leaves of WT *A. thaliana* Ler-0 plants compared to complete rosette leaves of *GLABRA1* plants. **e**-**f**, Comparison of gene expression levels of *GALT1* (**e**) and *FUT13* (**f**) in the trichomes and in complete rosette leaves of WT *A. thaliana* Col-0 plants compared to WT *A. thaliana* Ler-0 plants

F-box-Nictaba was identified as a functional lectin that can bind *N*- and *O*-glycans containing lactosamine structures, Lewis a, Lewis x and Lewis y motifs [18]. Arabidopsis plants were only reported to contain Lewis a-modified *N-*glycans. To our knowledge, the presence of Lewis a has not been studied yet in trichomes of *A. thaliana* plants. Since *F-box-Nictaba* expression is prominent in the trichomes, we addressed whether Lewis a motifs could be specifically synthesized in the trichomes. Therefore, qRT-PCR analysis was performed to analyse gene expression for the enzymes indispensable for Lewis a epitope synthesis in *A. thaliana*: i.e., *At1g26810* encoding β1,3-galactosyltransferase (GALT1) and *At1g71990* encoding α1,4-fucosyltransferase (FUT13) (Fig. 4) [21, 24, 26]. According to Fig. 4, both *GALT1* and *FUT13* are expressed in rosette leaves as well as in the leaf trichomes of *A. thaliana* Col-0 plants. The transcript levels for *GALT1* were significantly lower in the trichomes of WT *A. thaliana* Col-0 plants compared to those measured in the unprocessed rosette leaves of the same plants at all investigated developmental stages, the lowest value being a 5-fold decrease of *GALT1* expression in trichomes of 4-week-old plants (Fig. 4a). In 3- and 5-week-old plants *GALT1* mRNA levels were approximately two and three times lower in trichomes compared to rosette leaves, respectively (Fig. 4a). In contrast to *GALT1*, *FUT13* did not show a significant differential expression between trichomes and unprocessed rosette leaves (Fig. 4b).

Conversely, in trichomes of 3- and 4-week-old WT *A. thaliana* Ler-0 plants, the expression of the *GALT1* gene was markedly up-regulated by 7- and 29-fold, respectively, when compared to levels in unprocessed rosette leaves originating from these plants (Fig. 4c, white bars). Comparison of the *GALT1* expression levels in trichomes of WT *A. thaliana* Ler-0 plants to those in the trichomeless *GLABRA1* leaves revealed that *GALT1* mRNA levels are 5-, 20- and 7-fold higher in the trichomes, respectively, for the three plant stages tested (Fig. 4c, black bars). The differences in *FUT13* expression levels between trichomes and unprocessed rosette leaves from WT *A. thaliana* Ler-0 plants were smaller than for the *GALT1* gene expression. *FUT13* expression in trichomes of 4-week-old plants was only 4 times higher, whereas in 3- and 5-week-old plants there was no apparent difference in *FUT13* expression in trichome RNA and total RNA of Ler-0 leaf material (Fig. 4d, white bars). However, *FUT13* expression was 2.5-fold up-regulated in trichomes of *A. thaliana* Ler-0 plants compared to *GLABRA1* leaves of 4-week-old plants (Fig. 4d, black bars)*.* Similarly to the *F-box-Nictaba* expression pattern, *GALT1* and *FUT13* genes are more significantly up-regulated in the trichomes of 4-week-old plants compared to those of 3- and 5-week-old plants.

The *GALT1* gene was 3.5-7 times more expressed in the leaves of *A. thaliana* Col-0 plants compared to the corresponding leaves of *A. thaliana* Ler-0 plants (Fig. 4e, gray bars), with the highest differential expression in 4-week-old plants. In contrast, *GALT1* gene expression was 3-fold and 30-fold higher in the trichomes of 3- and 4-week-old *A. thaliana* Ler-0 plants compared to *A. thaliana* Col-0 plants (Fig. 4e, black bars). In general, WT *A. thaliana* Ler-0 plants had slightly higher transcript levels for *FUT13* than the WT *A. thaliana* Col-0 plants in unprocessed rosette leaves (Fig. 4f, gray bars). More important differences were observed in the trichomes of 3- and 4-week-old plants, where the *FUT13* transcript level was 2- and 5-fold higher in the *A. thaliana* plants with a Ler-0 background (Fig. 4f, black bars).

### Over-expression of *F-box-Nictaba* abrogates leaf damage and suppresses *Pst* DC3000 colonization at 4 and 5 dpi

Since *F-box-Nictaba* gene expression was significantly up-regulated after SA treatment (Fig. 1b) and infection with *Pst* DC3000 (Fig. 1d), it was suggested that F-box-Nictaba plays a role in plant defense responses. To investigate the relevance of F-box-Nictaba for plant resistance towards stress, transgenic *A. thaliana* Col-0 plants impaired in *F-box-Nictaba* gene expression (KO6 line) as well as plants with increased *F-box-Nictaba* gene expression (OE lines) (Additional file 1: Text S2) were subjected to infection with the virulent *Pst* DC3000 strain and assessed for disease symptoms and bacterial growth. The level of colonization 24 h post infection reflects the initial growth of *Pst* DC3000 within the apoplast, whereas growth of the pathogen in the next days after infection is accompanied by host programmed cell death and necrosis. Beforehand, transgenic F-box-Nictaba plants were proven to be morphologically indistinguishable from WT plants throughout growth and development (Additional file 1: Text S2).

**Fig. 5:**
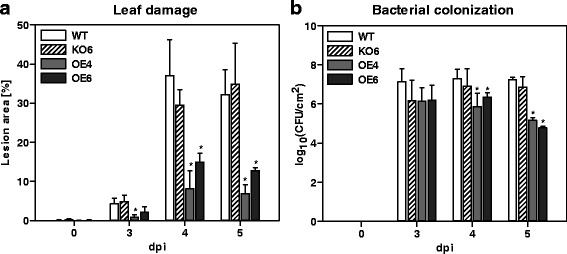
Development of disease symptoms and pathogen growth on the leaves of WT and transgenic KO6, OE4 and OE6 *A. thaliana* plants infected with *Pst* DC3000. **a**, Disease symptoms on leaves of infected *A. thaliana* plants measured as percent of lesion area. **b**, Pathogen growth in infected leaves. Levels of colonization are scored as CFU cm^−2^. The analysis was performed for two independent biological experiments, each with two technical replicates; error bars ± SE; *p < 0.05; **p < 0.01

As demonstrated in Fig. 5a and in Additional file 1: Figure S6, KO6 plants developed similar disease symptoms to WT plants, characterized by leaf lesions constituting up to 30 and 35 % of total leaf area at 4 and 5 dpi, respectively. Plants of both OE lines clearly exhibited less disease symptoms than the WT plants at 4 and 5 dpi, with lesions covering 8 to 12 % of total leaf area. Also at 3 dpi, OE4 plants demonstrated reduced disease symptoms compared to the WT plants. Consistent with the reduced levels of leaf damage, there was no significant difference in colonization of WT and KO6 plants whereas both OE lines yielded significantly lower bacterial counts compared to WT samples at 4 dpi and at 5 dpi (Fig. 5b).

**Fig. 6:**
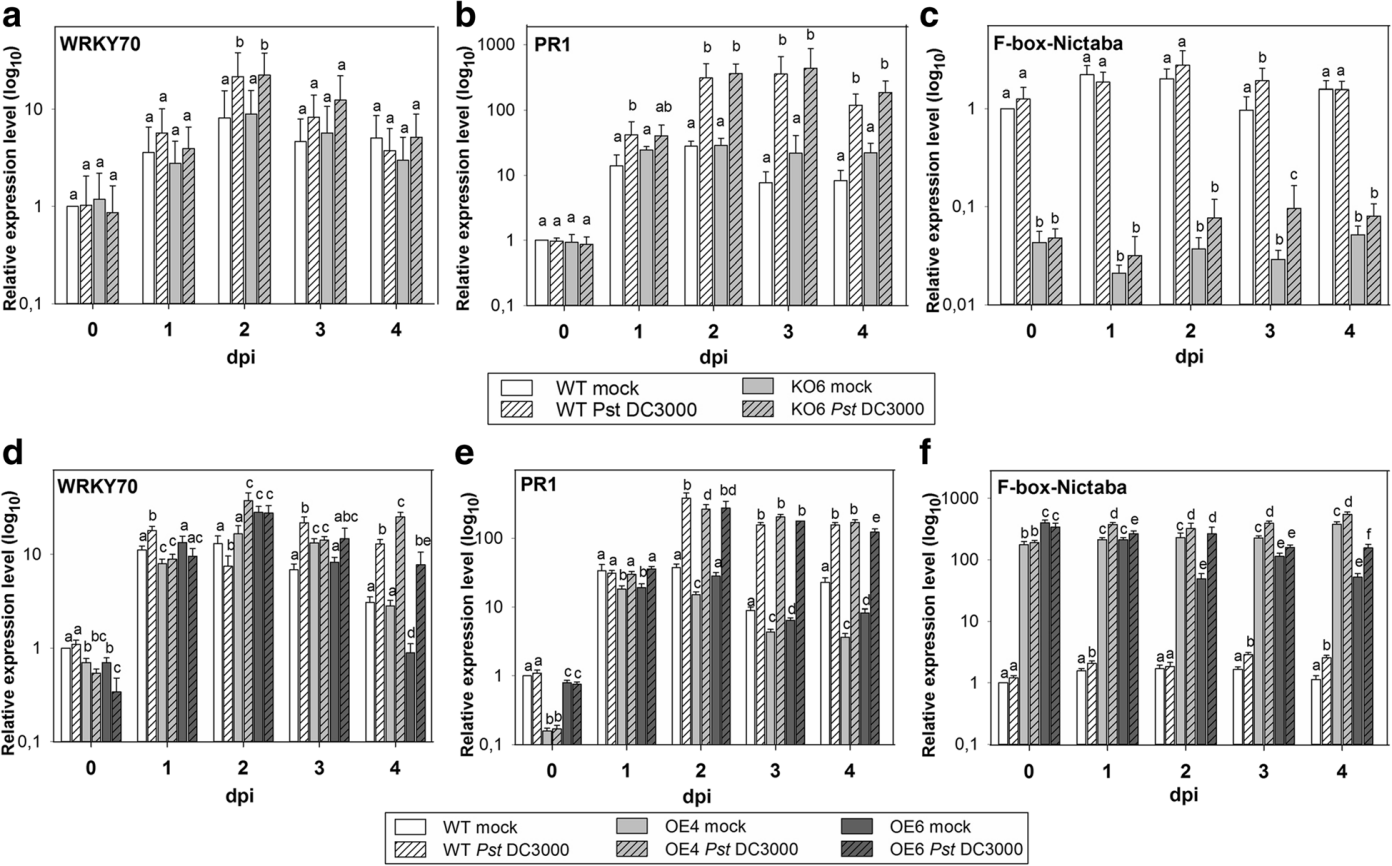
Transcript levels of *At2g02360* and positive control genes determined by qRT-PCR in 5-week-old transgenic *A. thaliana* plants after *Pst* DC3000 infection. **a** and **d**, Expression of *At3g56400* (WRKY70) in the KO6 line (**a**) and in lines OE4 and OE6 (**d**). **b** and **e**, Expression of *At2g14610* (PR1) in the KO6 line (**b**), and in lines OE4 and OE6 (**e**). **c** and **f**, Expression of *At2g02360* in the KO6 line (**c**), and in lines OE4 and OE6 (**f**). Gene expression levels were calculated relative to the expression in mock-treated WT plants at 0 dpi. For each time point different letters indicate statistically significant differential expression between different lines and compared to mock-treated plants (*p* < 0.05). Values were obtained from three independent biological experiments, each with three technical replicates; error bars ± SE

qRT-PCR analysis demonstrated no differential expression for any of the two positive controls *WRKY70* and *PR1,* neither in infected and mock-treated KO6 plants (Fig. 6a-b) nor in OE4 and OE6 plants (Fig. 6d-e). *F-box-Nictaba* expression remained stable in all transgenic plants over the time course of the infection experiment, with very low transcript levels in the KO6 line and significant 100-200-fold over-expression in the two OE lines compared to WT plants (Fig. 6c and f).

## Discussion

The qRT-PCR data and the meta-analysis database search show that, although a basal expression of *At2g02360* is present throughout plant development, this *F-box-Nictaba* is a SA- and pathogen-inducible gene. Supported by the expression profile of the *WRKY70* gene and the promoter analysis of *At2g02360*, it is likely that the SA-inducible expression of *At2g02360* is under the control of (a) SA-dependent WRKY transcription regulator(s). Judging from the delay in maximal expression for the *F-box-Nictaba* gene in comparison to one of the key SA-dependent WRKY transcription factors it is suggested that *F-box-Nictaba* is a late SA-responsive gene. As demonstrated by the *Pseudomonas* infection assays, *At2g02360* expression does not play an essential role in pathogen defense. It was reported previously that the *A. thaliana* genome contains more than 20 homologous genes encoding *At2g02360*-like genes for which evidence of expression is available [17, 19]. All these genes show considerable amino acid sequence similarity*,* reaching levels of > 90 and > 40 % sequence similarity for the F-box domain and the Nictaba domain, respectively. It is likely that some of these genes show functional redundancy and could fulfill the same or at least a similar role, as also demonstrated during male gametogenesis [27]. Therefore, it is suggested that At2g02360 will rather act as a partner in a complex network of defense-related proteins. Since transcript levels for *F-box-Nictaba* were up-regulated in WT *A. thaliana* plants after SA treatment and *Pst* DC3000 infection but expression was not affected by MeJA/ethephon treatment or infection with *B. cinerea,* it is reasonable to conclude that F-box-Nictaba plays a role in SA-mediated plant defense reactions to pathogen infection.

F-box-Nictaba expression is not only responsive towards biotic stresses, it is also up-regulated up to 3-fold after heat stress. Cross-talk between biotic and abiotic stress signaling pathways is quite common [28, 29]. *F-box-Nictaba* up-regulation upon heat stress might be driven by heat shock-activated transcription factors which can bind to the heat shock-responsive *cis-*elements present in the *At2g02360* promoter sequence, but also by heat stress-induced WRKY transcription factors activating *F-box-Nictaba* expression through the multiple W-box motifs present in its promoter sequence. Expression analysis of the *F-box-Nictaba* gene in *WRKY-*specific mutant lines could clarify the mode of transcriptional regulation of *F-box-Nictaba*.

In line with the plant defense-related expression of *At2g02360*, the histochemical assays showed preferential activity of the *At2g02360* promoter throughout plant development in non-glandular leaf trichomes of transgenic *A. thaliana* plants, especially in the trichomes present on young leaves. Similarly the qRT-PCR assays demonstrated a dominant presence of *At2g02360* mRNA in Arabidopsis leaf trichomes. Non-glandular trichomes are epidermal hairs on the surface of most plant aerial organs, characterized by a unicellular dendritic structure with stalk and three to four branches. They are implicated in transpiration control, thermotolerance and protection against insects, diseases and UV irradiation [30]. Although non-glandular trichomes are presumably non-secreting, they do express genes involved in the biosynthesis of secondary compounds suggesting their putative role in plant defense against pathogens [31]. In line with the presumed WRKY-dependent regulation of *F-box-Nictaba,* WRKY transcription factors are highly expressed in trichome tissues [32]. Alternatively, *F-box-Nictaba* gene expression in the trichomes may be under control of MYB transcription factors [33], which could bind to the multiple MYB-like *cis-*regulatory elements present in the *F-box-Nictaba* promoter and are reported to be responsible for trichome-specific gene regulation [34, 35]. Interestingly, two other F-box-Nictaba homologs are also predominantly expressed in trichomes [4]. One of these homologs, encoded by *At1g80110*, is up-regulated both in the trichomes and after infection with *Pst* DC3000.

The higher transcript levels for both *GALT1* and *FUT13* in the trichomes of WT *A. thaliana* with Ler-0 background strongly resembled the trichome-specific expression of the *F-box-Nictaba* gene under study. In general, all three genes are up-regulated more significantly in the trichomes of 4-week-old *A. thaliana* Ler-0 plants compared to those of 3- and 5-week-old plants. It is not entirely clear why the highest trichome-specific expression was reached at this particular time point, but the most probable explanation is that 4-week old *A. thaliana* plants are undergoing transition from the vegetative to the reproductive phase, characterized by stem formation and progression into bolting [36]. This is the time when major physiological changes are likely to occur and trichome production was shown to be affected significantly during this process [37–39]. Irrespective of the cause of the observed time-dependent differences, these data indicate that the two enzymes GALT1 and FUT13, required for biosynthesis of Lewis a structures, and the carbohydrate-binding protein F-box-Nictaba are co-expressed in the trichomes of *A. thaliana* Ler-0 plants. The fact that Lewis a containing glycans can be synthesized in the trichomes does not indicate that they are available to F-box-Nictaba within the nucleocytoplasmic compartment of the cell. Future studies will include interaction studies to clarify (1) the availability of Lewis a modified proteins in leaf trichomes and (2) the binding of the SA-induced F-box-Nictaba protein to these glycan structures.

## Conclusions

Detailed analysis of the transcript levels for the carbohydrate-binding F-box-Nictaba protein from *A. thaliana* demonstrated up-regulation after plant treatment with stress stimuli of both biotic and abiotic origin, including heat stress, the defense related plant hormone salicylic acid and after plant infection with the virulent *Pseudomonas syringae* pv. *tomato* strain DC3000. Furthermore, the histochemical assays show that the F-box-Nictaba gene is abundantly expressed in leaf trichomes, appendages involved in plant defense responses. Experiments with transgenic *A. thaliana* plants overexpressing the F-box-Nictaba protein demonstrated reduced disease symptoms after *Pst* DC3000 infection compared to the wild-type plants or plants in which F-box-Nictaba expression is reduced. Altogether the data strongly suggest that F-box-Nictaba is a defense protein involved in the plant response against *Pseudomonas* infection. Furthermore, we show that both F-box-Nictaba as well as the glycosyltransferases required for the synthesis of glycan motifs specifically recognized by F-box-Nictaba are co-expressed in the trichomes. Although the putative targets of F-box-Nictaba and the underlying mechanism of action remain to be elucidated, our research sheds a new light on the putative function of the non-glandular trichomes in plant responses to pathogen attack through glycan-based stress signaling.

## Methods

### Plant materials and culture conditions

Seeds of WT *A. thaliana* ecotype Col-0 were purchased from Lehle Seeds (Round Rock, Texas, USA). Seeds of WT *A. thaliana* ecotype Ler-0 (NW20), *GLABRA1* (N64) and SALK_085735C (herein renamed line KO6) [40] were obtained from the European Arabidopsis Stock Centre (NASC, University of Nottingham, UK). To establish in vitro cultures, surface sterilized seeds were sown on sterile filter paper which was placed on top of solid Murashige and Skoog (MS) medium (Duchefa, Haarlem, The Netherlands). To establish in vivo cultures, surface sterilized seeds were directly sown into artificial soil (Jiffy-7, 44 mm Ø) (AS Jiffy Products, Drobak, Norway), into expanded clay granules (>4 mm Ø) or in pot soil. To break dormancy, the seeds were stratified at 4 °C for three days in the dark. Afterwards, seeds were transferred to a controlled growth room set at 21 °C with a 16/8 h photoperiod for seed germination and plant development. Only for infection experiments and trichome isolation, seeds were kept in a growth chamber (Conviron Germany GmbH, Berlin, Germany) set at 21 °C with a 12/12 h photoperiod. All seedlings and plants were watered regularly. Plant samples were collected from different developmental stages as defined by [41]. Early stage plant materials (cotyledons, 4-leaves and 8-leaves stage) were collected from in vitro grown plants and included the complete seedlings, whereas the other samples were taken from plants grown in artificial soil. Root material was collected from plants grown in granules. Trichomes were isolated from rosette leaf material collected from plants grown in pot soil.

### Chemical reagents

BAP, GA_3_ and MeJA were purchased from Sigma-Aldrich (Bornem, Belgium). ABA and ethephon were obtained from Acros Organics (Geel, Belgium). SA, indole acetic acid (IAA) and salt were obtained from Duchefa, whereas mannitol was purchased from VWR (Leuven, Belgium). MG132 was obtained from Enzo Life Sciences (Antwerpen, Belgium). Prior to use, appropriate amounts of MeJA, SA, ABA, IAA and GA_3_ were dissolved in 100 % (w/v) ethanol, whereas BAP and MG132 were dissolved in 100 % (w/v) dimethyl sulfoxide (DMSO) (VWR). Ethephon, NaCl and mannitol were dissolved in water.

### Hormone treatments, abiotic stress applications and infection assays on WT *A. thaliana* plants

Hormone and abiotic stress treatments were performed according to [42–46] with modifications. Namely, in vitro grown 16-day-old seedlings were transferred from MS agar plates to Petri dishes filled with liquid MS medium containing either a hormone solution (100 μM concentration in case of ABA, BAP, ethephon, GA_3_, IAA or MeJA and 300 μM concentration in case of SA), 50 μM MG132, 150 mM NaCl or 100 mM mannitol and incubated at 21 °C for appropriate times. Controls were kept on liquid MS medium containing an equal volume of the corresponding solvent (ethanol, DMSO or water). Cold and heat stress were applied by incubating the MS agar plates with the seedlings in the dark either at 4 °C or 37 °C, respectively. Concomitantly, controls were incubated at 21 °C in the dark. For every stress application 20–30 seedlings were collected for RNA extraction at several time points, immediately frozen in liquid nitrogen and stored at −80 °C until use. Infection assays with *Pseudomonas syringae* pv. *tomato* strain DC3000 and *Botrytis cinerea* strain B05.10 were performed as described elsewhere [47–49]. For each infection, one hundred individually grown 5-week-old Arabidopsis plants were inoculated, either with infection or mock solution. Infection with *P. syringae* was performed by spraying the rosette leaves until run-off with bacteria from the mid to late log phase of growth (OD_600_ = 0.6–1.0), The inoculation solution consisted of bacteria resuspended in 10 mM MgSO_4_ supplemented with 0.05 % Silwet-77 (GE Specialty Materials (Suisse) S.a.r.l., Switzerland) to obtain a bacterial solution of OD_600_ = 0.05 corresponding to 2.5 × 10^7^ cfu / ml. One day before treatment up till two days after bacterial infection, the plants were maintained at 100 % relative humidity to increase the infection efficiency. Infection with *B. cinerea* was done by the droplet technique with spores harvested from 10-day-old cultures by putting a 10-μl droplet of spore solution on the upper side of three randomly chosen rosette leaves from each plant. The inoculation solution contained 5 × 10^5^ conidia / ml in ½ strength potato dextrose broth medium. In the fungal assay, the plants were kept at 100 % relative humidity during the entire experiment. At indicated time points post each infection, rosette leaves of 8–10 randomly chosen plants were frozen in liquid nitrogen and stored at −80 °C prior to use. All experiments for each hormone treatment, abiotic stress application and infection assay with *P. syringae* were performed with two independent biological experiments, and three technical replicates for each analysis. The infection experiment with *B. cinerea* was performed with two independent biological experiments, and two technical replicates.

### RNA extraction and cDNA synthesis

Plant samples were ground into a fine powder with a mortar and pestle and RNA extracted using TRI reagent (Sigma-Aldrich) according to the manufacturer’s instructions. To remove any residual genomic DNA, samples were treated with 2 units of RNase-free DNaseI (Fermentas, St. Leon-Rot, Germany) for 30 min at 37 °C. After addition of 2 μl EDTA (25 mM), the DNase enzyme was inactivated by incubation at 65 °C for 10 min. The RNA concentration and purity were measured with a Nanodrop 2000 Spectrophotometer (Thermo Scientific, USA). First-strand cDNA was synthesized from 1 μg of DNA-free total RNA with 1 μL of 50 μM oligo(dT)_20_ using the M-MLV transcriptase kit (Invitrogen) and then diluted 2.5x with RNase-free water. cDNA quality was checked by RT-PCR using primers specific for the *UBC9* gene (Additional file 1: Table S2).

### Quantitative RT-PCR assay

qRT-PCR analyses were performed using the SensiMix SYBR kit (Bioline Reagents Ltd, London, UK). The reaction mixture contained 1x SensiMix™ SYBR, 2 ng μL^−1^ first-strand cDNA and 500 nM of gene-specific forward and reverse primers (Additional file 1: Table S2) in a total volume of 20 μL. For hormone and stress treatments specific positive control genes were included according to [50–54]. qRT-PCR was carried out with 3 technical replicates in a Rotor-Gene 3000 (Corbett Life Science) using Rotor Discs (Qiagen, Hilden, Germany) as described [55].

### Construction of vectors for the GUS reporter system and for over-expression of F-box-Nictaba

The *pAt2g02360:GUS* reporter construct as well as the CaMV 35S:*At2g02360* construct were generated using the Gateway^TM^ cloning technology (Invitrogen). A 1806 nt *At2g02360* promoter fragment (including the 5′ UTR) was amplified by a two-step PCR starting from total genomic DNA extracted from WT *A. thaliana* Col-0 plants. The *At2g02360* coding sequence was amplified by a two-step PCR starting from the cDNA clone BX820545 (INRA, Centre de Toulouse, Unité de Recherche 1258-CNRGV, Castanet-Tolosan Cedex, France). In the first step, primers were used to generate an *At2g02360* promoter sequence (primers evd555 and evd556) and an *At2g02360* gene sequence (primers evd1046 and evd1047) including parts of the attB1 and attB2 Gateway adaptor sites at their 5′ and 3′ sequences, respectively (Additional file 1: Table S3). Primer sequences for promoter amplification were made based on *At2g02360* gene information as available on TAIR10. In the second step, primers evd2 and evd4 were used to complete the attB sites (Additional file 1: Table S3). AttB-PCR products were then cloned via the pDONR221 donor vector (Invitrogen) into the pKGWFS7.0 or pK7WG2.0 destination vector for promoter and gene sequence, respectively [56], sequenced and introduced into *A. tumefaciens* strain GV3101 using electroporation. WT Arabidopsis Col-0 plants were transformed using the floral-dip method [57]. Transgenic progenies were selected on MS agar plates supplemented with 75 mg L^−1^ kanamycin. Integration of the T-DNA into the plant genome was checked by PCR on genomic DNA using GUS-specific primers GUS-F and GUS-RV or kanamycin-specific primers evd463 and evd261 (Additional file 1: Table S3). Plants homozygous for the promoter:GUS construct in the T3 generation were used for the histochemical assays. Plants homozygous for the CaMV 35S:*At2g02360* construct (OE lines) in the T4 generation were used for infection experiments.

### Histochemical GUS assays

The GUS assay was performed according to [58]. Microscopic analysis was performed on a Nikon eclipse TE2000-e microscope (Nikon Belux, Brussels, Belgium) and a Leica DFC400 microscope (Leica, Heerbrugg, Germany) using the NIS-Elements and Leica Application Suite software packages, respectively.

### Isolation of leaf trichomes and subsequent RNA analysis

Leaf trichomes were isolated from rosette leaves of 3-, 4- and 5-week-old plants according to [59]. Purified trichomes were frozen in liquid nitrogen and stored at −80 °C until further processing. Yield, purity and integrity of the isolated trichomes was assessed by transmission microscopy using a Leica DFC400 microscope (Leica) with the Leica Application Suite software packages. For total RNA extraction, 1 mL TRI Reagent (Sigma-Aldrich) and 50 mg glass beads (300 μm Ø, Sigma-Aldrich) were added to approx. 60,000–80,000 trichomes (isolated from 25 g of rosette leaves). The solution was mixed at max speed (five cycles of 20 s mixing and 20 s rest on ice). For RNA extraction from unprocessed rosette leaves, 1 mL TRI Reagent was added to approx. 100 mg samples previously ground to a fine powder with a mortar and pestle. Trichome and complete leaf tissue extracts were then processed according to the manufacturer’s recommendations. DNase treatment, cDNA synthesis, RT-PCR and qRT-PCR were performed as described above. The complete experiment was repeated twice.

### Stress treatments of transgenic *A. thaliana* plants

Approximately sixty individually grown 5-week-old WT plants as well as transgenic *F-box-Nictaba-*specific KO and OE plants were inoculated with either the *Pst* DC3000 infection or the mock solutions as described above. At 0, 3, 4 and 5 dpi approximately hundred leaves were collected for each line and scanned with a flatbed scanner (Canoscan Lide 25). Scans of single leaves were processed with the ImageJ software. The damage to the leaves was determined as percent ratio of lesion area relative to the total leaf area using the disease assessment software of the APS Assess 2.0 program. Bacterial growth in the leaves of mock-treated and infected plants was determined by serial dilution assays as described [49]. Bacterial colonization was determined as colony forming units (CFU) cm^−2^ of sampled leaf tissue. RNA extraction, DNase treatment, cDNA synthesis and qRT-PCR for expression analysis at 0, 1, 2, 3, 4 and 5 dpi were performed as described above. The whole experiment for leaf damage assessment and bacterial colonization was performed with two independent biological experiments and three technical replicates for the each analysis.

## Additional file

Additional file 1: Figure S1.
*At2g02360* promoter sequence, with major putative *cis*-acting regulatory elements highlighted. **Figure S2.** Level of *At2g02360* expression during the lifecycle of WT *A. thaliana* Col-0 plants using the Genevestigator search tool. **Figure S3.** Relative transcript levels of selected positive control genes in stress-treated WT *A. thaliana* Col-0 plants. **Figure S4.** Relative transcript levels of *At2g02360* in 16-day-old WT *A. thaliana* Col-0 seedlings different stress treatments. **Figure S5.** *At2g02360* promoter sequence, with highlighted putative *cis*-acting regulatory elements possibly involved in promoter activity in trichomes. **Figure S6.** Development of disease symptoms on the leaves of WT and transgenic KO6, OE4 and OE6 *A. thaliana* plants infected with *Pst* DC3000. **Table S1.** Putative *cis*-acting regulatory elements identified with high frequency in the *At2g02360* promoter sequence by *in silico* analyses for identical motifs stored in the PLACE, PlantCARE and AGRIS databases. **Table S2.** Overview of all primers used in qRT-PCR. **Table S3.** Overview of all primers used in molecular cloning. **Text S1.** *In silico* expression analysis indicates that *At2g02360* is a stress-responsive gene. **Text S2.** Characterization of transgenic *A. thaliana* plants with altered F-box-Nictaba expression. (DOCX 1858 kb)

## Abbreviations

Dpi: Days post infection
ET: Ethylene
FBG: Glycan-binding F-box protein
FUT13: α1,4-fucosyltransferase
Gal: Galactose
GALT1: β1,3-galactosyltransferase
Glc: Glucose
GlcNAc: *N*-acetylglucosamine
JA: Jasmonic acid
KO: Knock-out
Le a: Lewis a structure
Nictaba: *Nicotiana tabacum* agglutinin
OE: Over-expression
*Pst* DC3000: *Pseudomonas syringae* pv. *tomato* strain DC3000
qRT-PCR: Quantitative reverse transcriptase polymerase chain reaction
SA: Salicylic acid
