# Supplementary material for: Glycan-binding F-box protein from Arabidopsis thaliana protects plants from Pseudomonas syringae infection
Source: BMC Plant Biol. 2016 Oct 4;16:213. doi: 10.1186/s12870-016-0905-2 (PMC5050601; doi:10.1186/s12870-016-0905-2)
Supplement: Additional file 1: Figure S1. — At2g02360 promoter sequence, with major putative cis-acting regulatory elements highlighted. Figure S2. Level of At2g02360 expression during the lifecycle of WT A. thaliana Col-0 plants using the Genevestigator search tool. Figure S3. Relative transcript levels of selected positive control genes in stress-treated WT A. thaliana Col-0 plants. Figure S4. Relative transcript levels of At2g02360 in 16-day-old WT A. thaliana Col-0 seedlings different stress treatments. Figure S5. At2g02360 promoter sequence, with highlighted putative cis-acting regulatory elements possibly involved in promoter activity in trichomes. Figure S6. Development of disease symptoms on the leaves of WT and transgenic KO6, OE4 and OE6 A. thaliana plants infected with Pst DC3000. Table S1. Putative cis-acting regulatory elements identified with high frequency in the At2g02360 promoter sequence by in silico analyses for identical motifs stored in the PLACE, PlantCARE and AGRIS databases. Table S2. Overview of all primers used in qRT-PCR. Table S3. Overview of all primers used in molecular cloning. Text S1. In silico expression analysis indicates that At2g02360 is a stress-responsive gene. Text S2. Characterization of transgenic A. thaliana plants with altered F-box-Nictaba expression. (DOCX 1858 kb) [file 12870_2016_905_MOESM1_ESM.docx]

**Additional file 1**

**Supplementary data Stefanowicz et al.**

**Supplemental Figures**

**Figure S1**

| 1 GGCCATCTTT TTAACCATTG CTGCATTTTG TTAAAAAACA AATCATATCT  51 ACAAGTCAGT GAGCTTGTAA AAAATGATCA TATGACATGA GAAAAAGAGA  101 AGTGAAGGGT TCTTACATGG AGGCGATCAC GGAACATAAG CCCGATTAAG  151 TAGGGGAGAA CCATGCCAAT GGTGGTTCCA ACCATGATAA TAACAAAACC  201 GAGACCATAA CCAAAAATCA TCCCAGCTAA CCACATGGAA GGACCAGAAG  251 GTATCAAGAA CACAGGAAAC AAAGCCAAGG AAACAACAAG GACAATCACG  301 AGCATAGGAC GGCCAAACGC AGTTGCTTCC CATTGCAAAA TTGGAATAAG  351 AACCTTCAAT AGAAACAGAC TATTACAATC CAAAGAGCAG AGTATGTGAC  401 ATGGATCACC ACAAGGAAAT AATCATCAGA AACTTGAGAT TTGAGTAAAG  451 AAATTACCTT TTGAAACACA AACGGAACTC CCCATTTTCC GAAAACAAGC  501 GTAAGCAAGA GAGCAACTGC ACATATTCCC AAAGCTTTTA ACCACCACAT  551 AAATTTCTTA CTCTGCACCT CTGATTGCGA TAGAGACAAC ACGGTTTCAG  601 CTGGGGAGGC TTCATGAGCC ACAACTAGCC GAACATACTC ATTATCCCTC  651 ATGTGAGGAG TAGAATTTGC AATATCCTCT CTTGACTCTT TCAATGGGTT  701 TGACATCAGT GTTCAGAACC TTAAAACTGA AGCCAAAATT CAATATCAGA  751 GACATAAAAT CACTATTTGT AAGCCATCAA AACAGGAAAA AATCACTGTT  801 CTATACATTA AACCAACCAA AACCGATCAA GGATAATGGA AACAAACCCT  851 AGAACATAGC TATATCAATA GATAAACAGA GAGATGATAA AGATGCTTAG  901 CTTAAAAATC AAAGTATTGA AAAAACTTCA GCAACTTGAA AGACACACAA  951 AGAGATTACA TACAAGTAAA CGATAACGAA AAAGTAAAGT TAATTACAGA  1001 AAGAATCAAA ACTCTCCGAA CATAGAAATT TCCGATTACG TCTAAAAAGG  1051 GGAAAATTCA AACCCAAAAT CAAACGATCA AATACATCAC TAGAGTAATC  1101 AAATCACTTC GATCTCTCAA TCTAGCTAAA GATCATCACA GGAATTATAA  1151 AGTAGTAGAG CTAGGGTTTA ACGAATCTCA CAAATCACAC ACTACGGAAA  1201 CCTAATCAAG CTTCTTGGTA TTAAACGACG GCGTTACGGC GGAGGAAGCT  1251 TGTACGCCGT CGGAAAAAAC TGGGTTGTTG CGGCGGATTT ACGTTTGTTT  1301 CAGGTTTAGT GTTGTTTTGC TAGCTTCTCA ACAAGAGAAG CGTGTTTTGT  1351 GTTTGTTTTT TTTCTTTAAT TCAAATGAAA AATAGATTGT TTGCTTTTAC  1401 TTAATCTCAT TTTTTATCAA TTTTTTCTTT CGTATTCGTT AGCTTAAGCT  1451 TTACTTTTCT GGAATAACGA GTCAGACTAT ATTATAAAAA TTCAAAACAA  1501 ATAATATTCC CACCGTTGAT CCACAATGCA TACCTGACTT GACCTTTTTT  1551 TAATTGGTTT GTTAGTTTGG AATTTGGATA TTTTCAAAAC CAAATCCTTA  1601 ACCAACGAAT CCACAATAAT GACCAAAAAA AAACAATGGA GCGTGAAGTA  1651 GACGCGTGGA TTGTTCACAA ATCAGGGCTA TTTTCGTCAA TATGTAGAAC  1701 GACTTGAAAT TTTCGTAATC CTTAGGGGTA CTTTCGTAAA TTAATAGAAC  1751 GACTTGAGAT TTTACACTAG CTTCCGTGGG AGACGGAGAC GAAGATACAA  1801 GCGTCG | | |
| --- | --- | --- |
| GATABOX | MYBIAT | GT1 motif |
| GT1CONSENSUS | MYBCCONSENSUSAT | TGA element |
| IBOXCORE | GT1GMSCAM4 | ARE |
|  |  |  |

**Figure S1**

*At2g02360* promoter sequence, with major putative *cis*-acting regulatory elements highlighted.

**Figure S2**

**
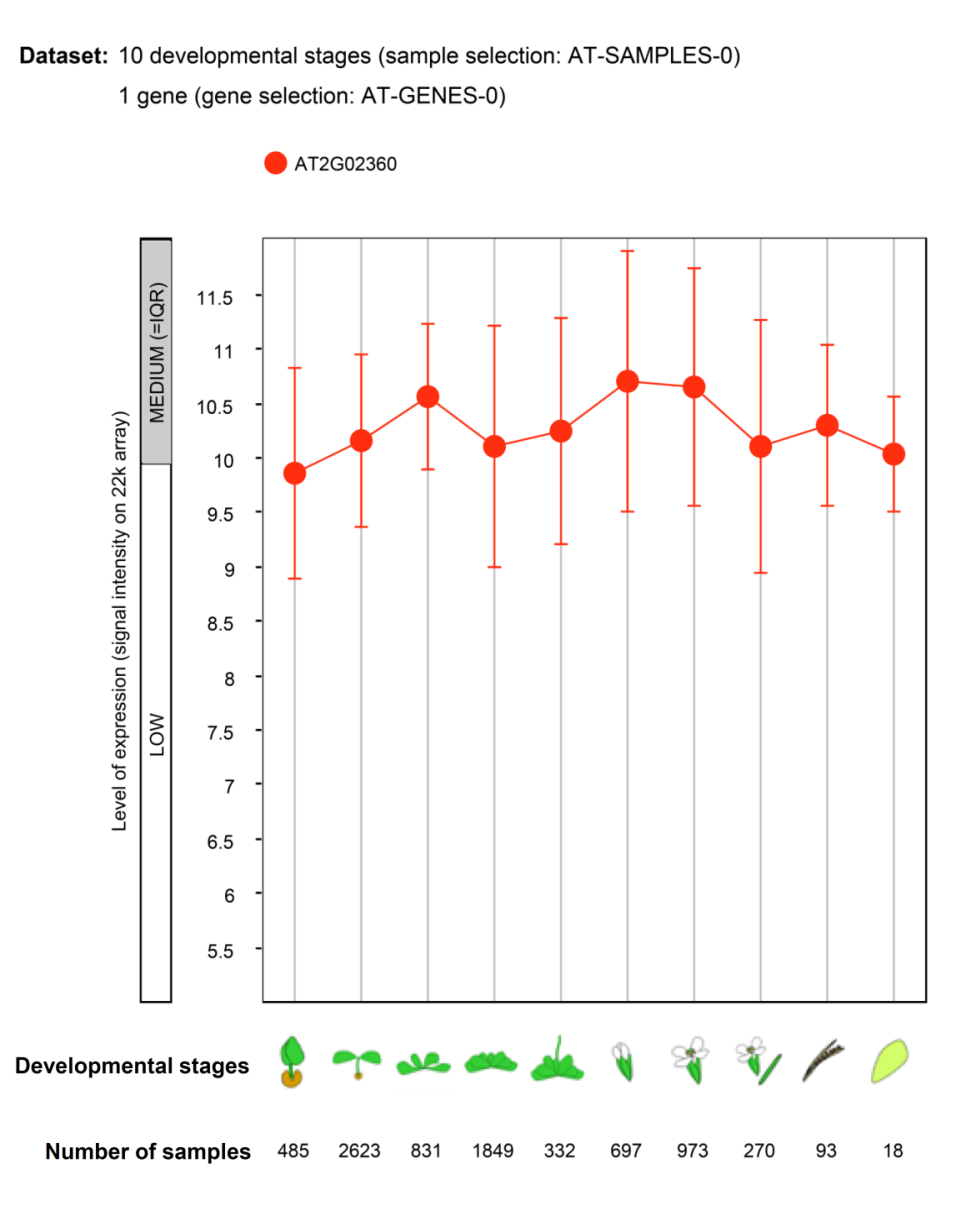
**

**Figure S2**

Level of *At2g02360* expression during the lifecycle of WT *A. thaliana* Col-0 plants using the Genevestigator search tool.

**Figure S3**

Figure S3

Relative transcript levels of selected positive control genes in stress-treated WT *A. thaliana* Col-0 plants determined by qRT-PCR analyses of two independent biological experiments, each with two technical replicates; error bars ± SE. Values are compared to gene expression levels determined in the mock-treated plants. Asterisks indicate statistically significant differential expression compared to control samples (*p<0.05; **p<0.01). (a), *At3g56400* (encoding WRKY70) expression levels in 16-day-old Arabidopsis seedlings after SA treatment. (b), *At1g16030* (encoding Hsp70b) expression levels in 16-day-old Arabidopsis seedlings after heat shock. (c), Relative expression of *At3g56400* (encoding WRKY70) and *At2g14610* (encoding PR1) after infection of 5-week-old Arabidopsis plants with *Pst* DC3000. (d), Relative expression of *At5g44420* (encoding PDF1.2) and *At2g14610* (encoding PR1) after infection of 5-week-old Arabidopsis plants with *B. cinerea* strain B05.10.

**Figure S4**

Figure S4

Relative transcript levels of *At2g02360* in 16-day-old WT *A. thaliana* Col-0 seedlings determined by qRT-PCR analyses of two independent biological experiments, each with two technical replicates; error bars ± SE. Asterisks indicate statistically significant differential expression compared to control samples (*p<0.05; **p<0.01). (a), *At2g02360* expression levels after treatment with 100 µM of plant hormones. (b), *At2g02360* expression levels after treatment with cold stress (4°C), 100 mM mannitol and 150 mM NaCl. (c), *At2g02360* expression levels in seedlings treated with 50 µM MG132.

Figure S5

| 1 GGCCATCTTT TTAACCATTG CTGCATTTTG TTAAAAAACA AATCATATCT  51 ACAAGTCAGT GAGCTTGTAA AAAATGATCA TATGACATGA GAAAAAGAGA  101 AGTGAAGGGT TCTTACATGG AGGCGATCAC GGAACATAAG CCCGATTAAG  151 TAGGGGAGAA CCATGCCAAT GGTGGTTCCA ACCATGATAA TAACAAAACC  201 GAGACCATAA CCAAAAATCA TCCCAGCTAA CCACATGGAA GGACCAGAAG  251 GTATCAAGAA CACAGGAAAC AAAGCCAAGG AAACAACAAG GACAATCACG  301 AGCATAGGAC GGCCAAACGC AGTTGCTTCC CATTGCAAAA TTGGAATAAG  351 AACCTTCAAT AGAAACAGAC TATTACAATC CAAAGAGCAG AGTATGTGAC  401 ATGGATCACC ACAAGGAAAT AATCATCAGA AACTTGAGAT TTGAGTAAAG  451 AAATTACCTT TTGAAACACA AACGGAACTC CCCATTTTCC GAAAACAAGC  501 GTAAGCAAGA GAGCAACTGC ACATATTCCC AAAGCTTTTA ACCACCACAT  551 AAATTTCTTA CTCTGCACCT CTGATTGCGA TAGAGACAAC ACGGTTTCAG  601 CTGGGGAGGC TTCATGAGCC ACAACTAGCC GAACATACTC ATTATCCCTC  651 ATGTGAGGAG TAGAATTTGC AATATCCTCT CTTGACTCTT TCAATGGGTT  701 TGACATCAGT GTTCAGAACC TTAAAACTGA AGCCAAAATT CAATATCAGA  751 GACATAAAAT CACTATTTGT AAGCCATCAA AACAGGAAAA AATCACTGTT  801 CTATACATTA AACCAACCAA AACCGATCAA GGATAATGGA AACAAACCCT  851 AGAACATAGC TATATCAATA GATAAACAGA GAGATGATAA AGATGCTTAG  901 CTTAAAAATC AAAGTATTGA AAAAACTTCA GCAACTTGAA AGACACACAA  951 AGAGATTACA TACAAGTAAA CGATAACGAA AAAGTAAAGT TAATTACAGA  1001 AAGAATCAAA ACTCTCCGAA CATAGAAATT TCCGATTACG TCTAAAAAGG  1051 GGAAAATTCA AACCCAAAAT CAAACGATCA AATACATCAC TAGAGTAATC  1101 AAATCACTTC GATCTCTCAA TCTAGCTAAA GATCATCACA GGAATTATAA  1151 AGTAGTAGAG CTAGGGTTTA ACGAATCTCA CAAATCACAC ACTACGGAAA  1201 CCTAATCAAG CTTCTTGGTA TTAAACGACG GCGTTACGGC GGAGGAAGCT  1251 TGTACGCCGT CGGAAAAAAC TGGGTTGTTG CGGCGGATTT ACGTTTGTTT  1301 CAGGTTTAGT GTTGTTTTGC TAGCTTCTCA ACAAGAGAAG CGTGTTTTGT  1351 GTTTGTTTTT TTTCTTTAAT TCAAATGAAA AATAGATTGT TTGCTTTTAC  1401 TTAATCTCAT TTTTTATCAA TTTTTTCTTT CGTATTCGTT AGCTTAAGCT  1451 TTACTTTTCT GGAATAACGA GTCAGACTAT ATTATAAAAA TTCAAAACAA  1501 ATAATATTCC CACCGTTGAT CCACAATGCA TACCTGACTT GACCTTTTTT  1551 TAATTGGTTT GTTAGTTTGG AATTTGGATA TTTTCAAAAC CAAATCCTTA  1601 ACCAACGAAT CCACAATAAT GACCAAAAAA AAACAATGGA GCGTGAAGTA  1651 GACGCGTGGA TTGTTCACAA ATCAGGGCTA TTTTCGTCAA TATGTAGAAC  1701 GACTTGAAAT TTTCGTAATC CTTAGGGGTA CTTTCGTAAA TTAATAGAAC  1751 GACTTGAGAT TTTACACTAG CTTCCGTGGG AGACGGAGAC GAAGATACAA  1801 GCGTCG | |
| --- | --- |
| AACCAAAC/GTTTGGTT | MYB-like recognition sites in direct and complementary strand |
| AACGTG/CACGTT | T/G-box elements in direct and complementary strand |

**Figure S5**

*At2g02360* promoter sequence, with highlighted putative *cis*-acting regulatory elements possibly involved in promoter activity in trichomes. Sequence analysis was performed with one mismatch allowed.

**Figure S6**

**
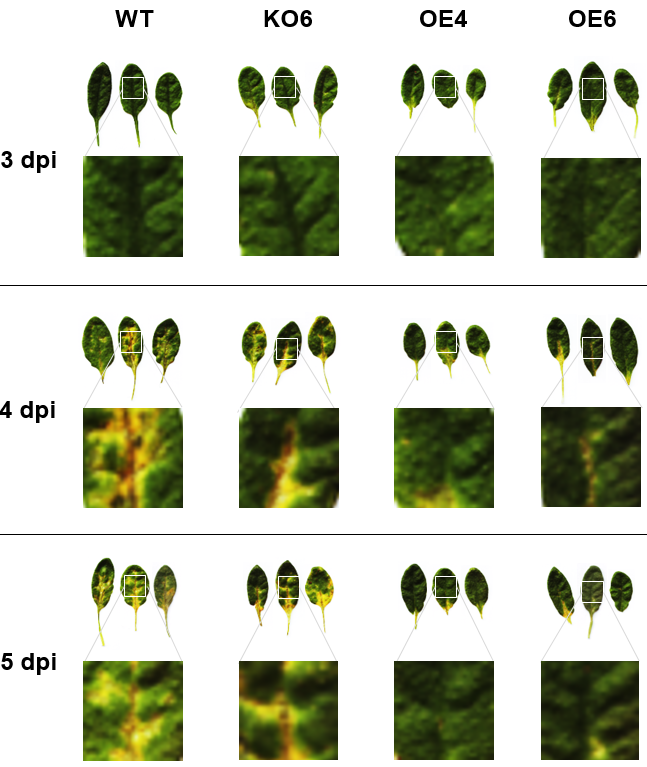
**

**Figure S6**

Development of disease symptoms on the leaves of WT and transgenic KO6, OE4 and OE6 *A. thaliana* plants infected with *Pst* DC3000. Photographs show representative rosette leaves collected from infected plants.

**Supplemental Tables**

**Table S1** Putative *cis*-acting regulatory elements identified with high frequency in the *At2g02360* promoter sequence by *in silico* analyses for identical motifs stored in the PLACE (part 1), PlantCARE (part 2) and AGRIS (part 3) databases.

| **Motif** | **Frequency** | **Description** |
| --- | --- | --- |
| **PART 1 – PLACE database output** | | |
| -300 ELEMENT | 3 | enhancer for endosperm specific-expression of glutenin |
| 2SSEEDPROTBANAPA | 1 | Important for napA promoter |
| AACACOREOSGLUB1 | 5 | endosperm-specific expression |
| ABRERATCAL | 1 | Ca^2+^-responsive element |
| ACGTATERD1 | 4 | Expression of erd1, induced by drought stress |
| AMYBOX1 | 3 | amylase box |
| ANAERO1CONSENSUS | 7 | motif in promoters of anaerobically induced genes |
| ANAERO3CONSENSUS | 2 | motif in promoters of anaerobically induced genes |
| ARFAT | 2 | response towards auxine |
| ARR1AT | 29 | ARR1 binding site |
| ASF1MOTIFCAMV | 1 | ASF-1 binding site |
| BIHD1OS | 3 | Binding site for transcription factor OsBIHD1 |
| BOXIINTPATPB | 4 | Important for NCII promoters |
| BOXLCOREDCPAL | 1 | Core sequence of box-L motif |
| CAATBOX1 | 21 | Tissue specific promoter element of legA gene in pea |
| CACTFTPPCA1 | 26 | Key component of Mem1 |
| CANBNNAPA | 1 | endosperm-specific expression |
| CATATGGMSAUR | 2 | response towards auxine |
| CCA1ATLHCB1 | 1 | Response towards light |
| CCAATBOX1 | 3 | motif in promoters of heat shock proteins |
| CGACGOSAMY3 | 3 | motif in GC-rich regions of rice amylase genes |
| CGCGBOXAT | 2 | Calmodulin-binding domain |
| CIACADIANLELHC | 1 | Necessary for Lhc circadian expression in tomato |
| CURECORECR | 4 | Copper and oxygen responsive element |
| DOFCOREZM | 26 | Binding site for Dof proteins |
| DPBFCOREDCDC3 | 2 | bZIP transcription factor, induced by ABA and embryo-specific |
| DRE1COREZMRAB17 | 1 | response towards ABA |
| E2FCONSENSUS | 1 | E2F consensus sequence |
| EBOXBNNAPA | 14 | E-box |
| EECCRCAH1 | 2 | Consensus motif for enhancer elements EE-1 and EE-2 |
| ELRECOREPCRP1 | 1 | response towards elicitors |
| ERELEE4 | 3 | response towards ET |
| GAREAT | 3 | response towards GA |
| GATABOX | 15 | response towards light and tissue-specific expression |
| GT1CONSENSUS | 26 | GT-1 binding site in light-induced genes |
| GT1CORE | 3 | Involved in binding of GT-1 to box II |
| GT1GMSCAM4 | 8 | Involved in pathogen and salt-induced gene expression |
| GTGANTG10 | 15 | Involved in expression of the late pollen gene g10 |
| HEXAMERATH4 | 2 | motif in histon H4 promoter of *A. thaliana* |
| IBOXCORE | 7 | response towards light |
| INRNTPSADB | 4 | initiator in promoters of genes in tobacco lacking a TATA-box |
| LTRE1HVBLT49 | 1 | Response towards low temperature |
| LTRECOREATCOR15 | 1 | core of LTRE-1 |
| MYB1AT | 8 | response towards drought |
| MYB1LEPR | 1 | Involved in defense regulated gene expression in tomato |
| MYB2CONSENSUSAT | 3 | response towards drought |
| MYBATRD22 | 1 | response towards drought |
| MYBCORE | 3 | response towards water stress |
| MYBCOREATCYCB1 | 2 | Involved in activation of receptor genes |
| MYBGAHV | 3 | response towards GA |
| MYBPLANT | 1 | MYB binding site |
| MYBPZM | 2 | MYB binding site |
| MYBST1 | 4 | MYB binding site |
| MYCATERD1 | 2 | response towards drought |
| MYCATRD22 | 2 | response towards drought |
| MYCCONSENSUSAT | 14 | response towards drought |
| NODCON1GM | 3 | Noduline sequence |
| NODCON2GM | 7 | Noduline sequence |
| NTBBF1ARROLB | 2 | Tissue specific expression, response towards auxine |
| OSE1ROOTNODULE | 3 | Active in infected cells of root nodules |
| OSE2ROOTNODULE | 7 | Active in infected cells of root nodules |
| PALBOXAPC | 1 | Present in fenylalanine ammoniumlyase genes |
| POLASIG2 | 2 | plant polyA signal |
| POLASIG3 | 3 | plant polyA signal |
| POLLEN1LELAT52 | 10 | Involved in pollen-specific activation of tomato genes |
| PREATPRODH | 1 | pro-osmolarity responsive element |
| PRECONSCRHSP70A | 7 | consensus sequence of the pro-osmolarity responsive element |
| PYRIMIDINEBOXHVEPB1 | 2 | pyrimidine-box |
| PYRIMIDINEBOXOSRAMY1A | 3 | pyrimidine-box |
| QELEMENTZMZM13 | 1 | Involved in enhancer activity |
| RAV1AAT | 5 | Binding site for transcription factors in *A. thaliana* |
| RBCSCONSENSUS | 1 | rbcS consensus sequence |
| REALPHALGLHCB21 | 6 | Involved in phytochrome regulation |
| RHERPATEXPA7 | 1 | Root hair-specific *cis*-element |
| ROOTMOTIFTAPOX1 | 8 | motif in promoter of rolD gene |
| SEBFCONSSTPR10A | 1 | response towards auxine |
| SEF3MOTIFGM | 3 | SEF3 binding site (soybean embryo factor) |
| SEF4MOTIFGM7S | 3 | SEF4 binding site (soybean embryo factor) |
| SORLIP1AT | 1 | response towards light |
| SREATMSD | 2 | Sugar-repressive element |
| SURECOREATSULTR11 | 3 | sulfur-responsive element |
| SV40COREENHAN | 2 | SV40 core enhancer |
| TAAAGSTKST1 | 7 | TAAG-motif |
| TATABOX3 | 1 | TATA-box |
| TATABOX5 | 2 | TATA-box |
| TATCCAOSAMY | 1 | element present in α-amylase promoters |
| TBOXATGAPB | 1 | T-box in promoter of GAPB gene |
| TGTCACACMCUCUMISIN | 1 | enhancer involved in fruit-specific expression of cucumisin |
| TRANSINITDICOTS | 1 | Initiation codon for translation in dicots |
| TRANSINITMONOCOTS | 1 | Initiation codon for translation in monocots |
| UP2ATMSD | 2 | Up2 motif |
| VOZATVPP | 2 | VOZ-binding site involved in pollen development |
| WBOXATNPR1 | 4 | W-box |
| WBOXHVISO1 | 4 | W-box |
| WBOXNTCHN48 | 3 | W-box |
| WBOXNTERF3 | 6 | W-box |
| WRKY710S | 10 | W-box |
| **PART 2 – PlantCARE database output** | | |
| G-box | 1 | response towards light |
| GT1 motif | 4 | response towards light |
| MRE | 1 | response towards light |
| TCCC motif | 1 | response towards light |
| TCT motif | 2 | response towards light |
| chs-CMA1a | 2 | response towards light |
| AE-box | 2 | response towards light |
| Box I | 2 | response towards light |
| GA motif | 1 | response towards light |
| GAG motif | 1 | response towards light |
| GATA motif | 1 | response towards light |
| LAMP-element | 1 | response towards light |
| Sp1 | 1 | response towards light |
| as-2-box | 1 | response towards light |
| Box 4 | 1 | response towards light |
| Gap-box | 1 | response towards light |
| Box-W1 | 1 | Elicitor responsive element |
| ELI-box3 | 1 | Elicitor responsive element |
| GARE motif | 1 | response towards GA |
| P-box | 1 | response towards GA |
| TGA-element | 3 | response towards auxine |
| CGTCA motif | 1 | MeJA-responsive element |
| TGACG motif | 1 | MeJA-responsive element |
| TCA-element | 2 | response towards SA |
| HSE | 1 | Heat stress element |
| LTR | 1 | Involved in cold response |
| MBS | 2 | Response towards drought stress |
| ARE | 3 | Essential for anaerobic induction |
| TC-rich repeats | 1 | Involved in defense and stress response |
| O2-site | 1 | Involved in zein metabolism |
| Unnamed_6 | 1 | SEF4 binding site |
| CCAAT-box | 1 | MYBHv1 binding site |
| Skn-1_motif | 2 | Involved in endosperm-specific expression |
| CAAT-box | 36 | Promoter element |
| TATA-box | 30 | Promoter element |
| CCGTCC-box | 1 | Involved in meristem specific activation |
| circadian | 1 | Involved in expression of circadiana genes |
| A-box | 1 | Function unknown |
| AAGAA-motif | 3 | Function unknown |
| Box E | 1 | Function unknown |
| CTAG-motif | 1 | Function unknown |
| W-box | 1 | Binding site for WRKY transcription factors |
| **PART 3 – AGRIS database output** | | |
| W-box promoter motif | 1 | Binding site for WRKY transcription factors |
| DPBF1 and 2 binding site motif | 1 | Binding site for bZIP transcription factors |
| MYB4 binding site motif | 1 | Binding site for MYB4 transcription factor |
| LFY consensus binding site motif | 1 | Binding site for LFY transcription factor |
| BOXII promoter motif | 1 | Function unknown |

**Table S2** Overview of all primers used in qRT-PCR.

| **Target gene** | **Forward primer (5’-3’)** | | **Reverse primer (5’-3’)** | |
| --- | --- | --- | --- | --- |
| *F-box-Nictaba* gene *(At2g02360)* | evd786 | TTGAGCTTGGGGAGTTCTTC | evd787 | AGAGGATTTTAGCAGGTCGG |
| *GALT1 ( At1g26810)* | evd1153 | AGTGATGGATGCAAGGATGG | evd1154 | GAGAGCGTTTGGTTTCTTGG |
| *FUT13 (* *At1g71990)* | evd1155 | TTTCTATGCGCTCGACTCTG | evd1156 | GAGCCGAATTTGCTACCATC |
| *PP2A* (*At1g13320) –* reference gene for data normalization | evd727 | TCCGAGATCACATGTTCCAAACTC | evd728 | CCGTATCATGTTCTCCACAACCG |
| *TIP41 (At4g34270) –* reference gene for data normalization | evd729 | TGAACTGGCTGACAATGGAGTG | evd730 | CATGAGCTTGGCATGACTCTCAC |
| *UBC9 (At4g27960) –* reference gene for data normalization | evd731 | TCCTACTTCATGTAGCGCAGGAC | evd732 | TCCTCCAGAATAAGGGCTATCCG |
| *ARR5 (At3g48100) –* positive control for BAP treatment | evd741 | CCTGATTCTTTCGGCTTACAATTT | evd742 | TGATCAGTCTTGGTTCTATCAGCAA |
| *COR15A (At2g42540) –* positive control for ABA and cold treatment | evd781 | CAGTGAAACCGCAGATACATTGGG | evd782 | GGCTTCTTTTCCTTTCTCCTCC |
| *ERS1 (At2g40940) –* positive control for ethephon treatment | evd813 | GGTTTGTCGGGCTAATGG | evd814 | ACCACTGCTACTGCTTGGAC |
| *GAI (At1g14920) –* positive control for GA_3_ treatment | evd743 | AATGAATTGATCTGTTGAACCGG | evd744 | GGCTTCGGTCGGAAATCTATC |
| *HsfA2* *(At2g26150) –* positive control for MG132 treatment | evd1095 | gtgttgaggttgggcaatacg | evd1096 | ttgctgttgcctcaacctaactac |
| *Hsp70b* *(At1g16030) –* positive control for heat treatment | evd735 | ATGTATCAGGGTGGTGCTGCT | evd736 | ACCTCTTCGATCTTGGGACCT |
| *IAA1 (At4g14560) –* positive control for IAA treatment | evd739 | AGGACACAGAGCTTCGTTTGG | evd740 | GTCGTTGTTCTTGCGCTTGT |
| *JMT (At1g19640) –* positive control for MeJA treatment | evd745 | TATGTAAGCTCGCCACGATACGCT | evd746 | AACACGATCAACCGGCTCTAACGA |
| *PDF1.2 (At5g44420) –* positive control for *B. cinerea* infection | evd788 | AAGTTGTGCGAGAAGCCAAG | evd789 | CCATGTTTGGCTCCTTCAAG |
| *PR1 (At2g14610) –* positive control for *Pseudomonas* infection | evd1019 | GCTACGCAGAACAACTAAGAGG | evd1020 | GCCTTCTCGCTAACCCACAT |
| *RD29A (At5g52310) –* positive control for mannitol and NaCl treatment | evd749 | ATCACTTGGCTCCACTGTTGTTC | evd750 | ACAAAACACACATAAACATCCAAAGT |
| *WRKY70 (At3g56400) –* positive control for SA treatment and *Pseudomonas* infection | evd811 | CATGGATTCCGAAGATCACA | evd812 | CTGGCCACACCAATGACAA |

**Table S3** Overview of all primers used in molecular cloning.

| **Target gene/sequence** | **Forward primer (5’-3’)^a^** | **Reverse primer (5’-3’)^a^** |
| --- | --- | --- |
| 1806 nt promoter sequence of *At2g02360* | evd 555  AAAAAGCAGGCTTCGGCCATCTTTTTAACCATTGC | evd 556  AGAAAGCTGGGTGCGACGCTTGTATCTTCGTC |
| full-length *F-box-Nictaba* (*At2g02360*) gene sequence | evd1046  AAAAAGCAGGCTTCACCATGgggagaaaacgcaga | evd1047  AGAAAGCTGGGTGTCAgaggattttagcaggtcgg |
| attB1 and attB2 adaptor sites | evd 2  GGGGACAAGTTTGTACAAAAAAGCAGGCT | evd 4  GGGGACCACTTTGTACAAGAAAGCTGGGT |
| *NptII* (kanamycine resistance gene) | evd 463  GAACAAGATGGATTGCACGCAGG | evd 261  TCAGAAGAACTCGTCAAGAAGGCG |
| *UidA* (β-glucuronidase gene) | GUS-F  AAAAAGCAGGCTTCGATTTGGAAACGGCAGAGAAGG | GUS-RV  AGAAAGCTGGGTGTTTCTTGTTACCGCCAACGCG |

^a^ Nucleotides underlined are complementary to parts of the attB1 and attB2 gateway cloning sites.

**Text S1 *In silico* expression analysis indicates that *At2g02360* is a stress-responsive gene.**

**Methods**

Promoter sequences were scanned for *cis*-acting regulatory elements using the PlantCARE [1] and PLACE [2] databases and the AGRIS information server [3]. A transcriptome analysis was performed using TBLASTN for *A. thaliana*. Large scale expression data sets and microarray data as available on the eFP browser [4] were analyzed to obtain a hypothetical expression dataset for the *At2g02360* gene in *A. thaliana* cells and tissues.

**Results**

The PLACE, PlantCARE and AGRIS databases contain both experimentally validated and predicted information about upstream regions of annotated *A. thaliana* genes including *cis*-regulatory elements and transcription factor binding sites [1-3]. To obtain information related to the presence of gene regulatory *cis*-elements in the *F-box-Nictaba* promoter sequence, an *in silico* survey was performed by scanning these databases with the 1800 nt upstream region of the *At2g02360* gene as the input sequence (see Supplemental Fig. S1). As such, the regular core promoter elements, *i.e.* the CAAT box, the CCAAT box and the TATA box, were found 15, 3 and 3 times respectively, indicating that our input sequence encoded a promoter sequence.

Further analysis for tissue or stress-related elements yielded a list of many putative *cis*-regulatory elements related to responsiveness towards hormonal stress as well as abiotic stress signaling (see Supplemental Table S1). According to both the PLACE and PlantCARE databases light-responsive elements such as GATA boxes, CT1CONSENSUS and I boxes constituted the largest group, followed by putative drought stress-responsive elements such as MYB1AT and MYCCONSENSUSAT, a salt-responsive element and many hormone-responsive elements, including elements responsive towards auxin, abscisic acid (ABA), salicylic acid (SA), gibberellic acid (GA_3_) and ethylene. The AGRIS survey resulted in a much shorter list of putative *cis*-elements including only the SA-responsive W-box element (binding site for WRKY transcription factors), an ABA-responsive DPBF1&2 binding site, a MYB4 binding site (indicative for environmental stress response) and a LFY consensus site.

Screening the expression dataset of *At2g02360* as present in the thousands of microarray experiments ensembled on the eFP browser [4] and Genevestigator tool [5] revealed that during normal development of *A. thaliana* *At2g02360* was seemingly more expressed in rosette and cauline leaves compared to other tissues of the plant (see Supplemental Fig. S2). Interestingly, upon heat stress (38°C) and SA treatment (10 µM) the *At2g02360* gene was up-regulated two-fold in all plant tissues. Similarly, mannitol (300 mM) treatment resulted in two-fold elevated *At2g02360* expression levels, more specifically in the leaves. Infection with a virulent *Pseudomonas* species also enhanced *At2g02360* expression. In contrast, *At2g02360* expression was two times down-regulated after salt treatment. Also a transcriptome analysis by TBLASTN searches against the *A. thaliana* EST database indicated expression of *At2g02360* upon various hormone and abiotic stress treatments (Results not shown).

**References**

**[1]** Lescot M, Déhais P, Thijs G, Marchal K, Moreau Y, Van de Peer Y, Rouzé P, Rombauts S. PlantCARE, a database of plant *cis*-acting regulatory elements and a portal to tools for *in silico* analysis of promoter sequences. Nucleic Acids Res. 2002;30:325-327.

**[2]** Higo K, Ugawa Y, Iwamoto M, Korenaga,T. Plant cis-acting regulatory DNA elements (PLACE) database: 1999. Nucleic Acids Res.1999;27:297-300.

**[3]** Yilmaz A, Mejia-Guerra MK, Kurz K, Liang X, Welch L, Grotewold E. AGRIS: Arabidopsis gene regulatory information server, an update. Nucleic Acids Res. 2011;39:D1118-D1122.

**[4]** Winter D, Vinegar B, Nahal H, Ammar R, Wilson GV, Provart NJ. An “Electronic Fluorescent Pictograph” browser for exploring and analyzing large-scale biological data sets. Plos One 2007;2:e718.

**[5]** Hruz T, Laule O, Szabo G, Wessendorp F, Bleuler S, Oertle L, Widmayer P, Gruissem W, Zimmermann P. Genevestigator V3: a reference expression database for the meta-analysis of transcriptomes. Adv in Bioinformatics 2008;420747:doi:10.1155/2008/420747.

**Text S2 Characterization of transgenic *A. thaliana* plants with altered F-box-Nictaba expression**

**Methods**

**Plant materials and growth conditions**

Seeds of WT *A. thaliana* Col-0 were purchased from Lehle Seeds (Round Rock, Texas, USA). Seeds of SALK T-DNA insertion mutant lines associated with the *At2g02360* locus SALK_007866 and SALK_085735C (further referred to as knockout lines KO4 and KO6, respectively) [1], were obtained from the European Arabidopsis Stock Centre (NASC, University of Nottingham, UK). To establish *in vitro* cultures, surface sterilized seeds were sown on sterile filter paper which was placed on top of solid Murashige and Skoog (MS) medium (Duchefa, Haarlem, The Netherlands). To break dormancy, the seeds were stratified at 4°C for three days in the dark. Afterwards, seeds were transferred to a controlled growth chamber set at 21°C with a 16/8 h light/dark photoperiod for seed germination and plant development. A cell suspension culture of WT *A. thaliana* was maintained as described [2].

**Selection of SALK lines with KO expression of *At2g02360***

Two lines with a T-DNA insertion site within the exon sequences of the *At2g02360* locus were retrieved from the SALK database ([http://signal.salk.edu/cgi-bin/tdnaexpre​ss](http://signal.salk.edu/cgi-bin/tdnaexpress)). Both lines were selected on MS agar plates supplemented with 75 mg L^-1^ kanamycin (Duchefa) and tested for homozygosity by PCR on total genomic DNA. For each SALK line a different set of three primers was used, including the left border primer of the T-DNA insertion (LBb1.3) as well as two line-specific primers, *i.e.* the left (LP) and the right genomic primer (RP). For each line, two PCR reactions were set up with the primer combinations “LP+RP” and “LBb1.3 + RP”. Identical PCR reactions were performed on genomic DNA extracted from *A. thaliana* WT plants. The DNA quality was checked by PCR using *actin2* specific primers. Gene expression of *F-box-Nictaba* was analyzed by RT-PCR using primers evd 790 and evd 791 for amplification of the full-length sequence. cDNA quality was checked using *PP2A* specific primers. qRT-PCR using the *F-box-Nictaba* specific primers evd786 and evd787 was performed to quantify gene expression levels.

**Primer sequences**

UBC9-F (evd 731): 5’TCCTACTTCATGTAGCGCAGGAC3’

UBC9-R (evd 732): 5’TCCTCCAGAATAAGGGCTATCCG 3’

LBb1.3 (P99): 5’ ATTTTGCCGATTTCGGAAC3’

LP-KO4 (evd 1009): 5’CAGGCAACGAATCGAGAGTAG3’

RP-KO4 (evd 1010): 5’AAACTTCGCGATGTATGTTGG3’

LP-KO6 (evd 1011): 5’AATCTCCATCCACCCATCTTC3’

RP-KO6 (evd 1012): 5’GTAGACGCGTGGATTGTTCAC3’

ACT2-F (evd 280): 5’ GGCTGGATTTGCTGGAGATGATGC3’

ACT2-R (evd 281): 5’ GTACGACCACTGGCATACAGGGA3’

At2g02360-F full-length (evd790): 5’ CACCATGGGGAGAAAACGCAGAG3’

At2g02360-R full-length (evd791): 5’ TCAGAGGATTTTAGCAGGTCGG3’

PP2A-F (evd 727): 5’ TCCGAGATCACATGTTCCAAACTC3’

PP2A-R (evd 728): 5’ CCGTATCATGTTCTCCACAACCG3’

At2g02360-F (qPCR) (evd786): 5’ TTGAGCTTGGGGAGTTCTTC3’

At2g02360-R (qPCR) (evd 787): 5’ AGAGGATTTTAGCAGGTCGG3’

**RNA extraction, cDNA synthesis and RT-PCR analysis**

Leaf samples were ground into a fine powder with a mortar and pestle and RNA extracted using TRI reagent (Sigma-Aldrich) according to the manufacturer’s instructions. To remove any residual genomic DNA, samples were treated with 2 units of RNase-free DNaseI (Fermentas, St. Leon-Rot, Germany) for 30 min at 37°C. After addition of 2 µl EDTA (25mM), the DNase enzyme was inactivated by incubation at 65°C for 10 min. The RNA concentration and purity were measured with a Nanodrop 2000 Spectrophotometer (Thermo Scientific, USA). First-strand cDNA was synthesized from 1 µg of DNA-free total RNA with 1 µL of 50 µM oligo(dT)_20_ using the M-MLV transcriptase kit (Invitrogen) and then diluted 2.5x with RNase-free water. The cDNA quality was checked by RT-PCR using UBC9-F and UBC9-R primers, specific for the SUMO-conjugating enzyme *UBC9* gene.

**Quantitative RT-PCR (qRT-PCR) analysis**

qRT-PCR analyses have been performed using the SensiMix SYBR kit (Bioline Reagents Ltd, London, UK). The reaction mixture contained: 1x SensiMix™ SYBR, 2 ng μL^-1^ first-strand cDNA and 500 nM of gene-specific forward and reverse primers in a total volume of 20 μL. qRT-PCR was carried out in a Rotor-Gene 3000 (Corbett Life Science) using Rotor Discs (Qiagen, Hilden, Germany) as described by [3].

**Protein extraction, SDS-PAGE and Western blot analysis**

Protein samples were extracted by grinding leaf material or cells with a mortar and pestle in 1x PBS buffer containing 1mM PMSF and 1 µg mL^-1^ of pepstatin, leupeptin and aprotinin (all from Sigma). The resulting crude protein extracts were transferred to 1.5 ml eppendorf tubes and centrifuged at 4°C for 10 min at 12,000 rpm. Supernatants were collected for subsequent SDS-PAGE and Western blot analysis as described [4].

**Development of an anti-F-box-Nictaba antibody**

A peptide-based antibody specific against the F-box-Nictaba protein has been produced in a guinea pig production platform by the Thermo Scientific custom antibody service. Based on the analysis of the amino acid sequence of the F-box-Nictaba protein with the Antigen Profiler software (http://www.pierce-antibodies.com/custom-antibodies/peptide-design-antigen-profiler.cfm), the peptide ^201^cfseairrgrrnvvkpkqre^220^ was selected for immunization. Before and after immunization, sera of selected animals were tested by Western blot for reactivity against the recombinant F-box-Nictaba protein and its Nictaba domain as described [4], recombinant Nictaba from tobacco plants as described [5] as well as against total protein extracts from WT *A. thaliana* plants and cells.

**Germination rate assessment of transgenic *A. thaliana* plants**

Two hundred surface sterilized seeds of WT *A. thaliana* plants and of transgenic KO plants (impaired in *At2g02360* gene expression) and OE plants (overexpressing the *F-box-Nictaba* gene *At2g02360*) were directly sown on MS agar plates (25 seeds per line per plate). After three days of stratification, plates were transferred to a controlled growth chamber set at 21°C with a 16/8 h light/dark photoperiod and germinated seeds were counted daily for five days.

**Results**

**Line SALK_085735C (KO6) is a true KO mutant impaired in *F-box-Nictaba* gene expression**

The SALK population comprises single, segregating flank-tagged T-DNA insertion lines generated by Dr. Joseph Ecker (The Salk Institute in California, USA) via *A. tumefaciens* vacuum infiltration of *A. thaliana* Col-0 plants [1]. Based on the information retrieved from the SALK database, two SALK T-DNA lines have been selected with a T-DNA insertion in exon sequences of *At2g02360*, i.e. in SALK_007866 (KO4) and SALK_085735C (KO6) (Fig. ST1a). Plants of both KO4 and KO6 lines were tested for homozygosity by PCR on total genomic DNA using the left border primer of the T-DNA insertion (LBb1.3) as well as the line-specific left (LP) and right (RP) genomic primers (Fig. ST1b-c). By using gene-specific primers spanning the insertion site (primer combinations 1 and 3), PCR products of 1067 bp and 1060 bp expected for WT allele were amplified from WT DNA but not from mutant DNA. PCRs with the T-DNA-specific primer and RP gene-specific primers (primer combinations 2 and 4) showed products in the range of 700 bp for mutant DNAs, but not for the WT line (Fig. ST1b). This is in good agreement with the expected products for mutant alleles of 795 bp and 746 bp for KO4 and KO6, respectively. Thus, both tested SALK T-DNA insertion lines are homozygous.

| **(a)**  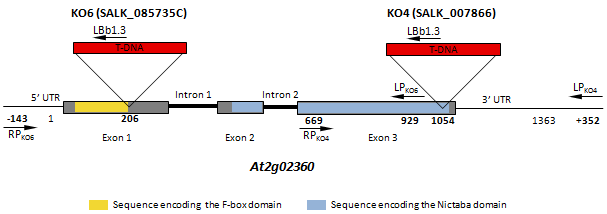 | |
| --- | --- |
| **(b)**  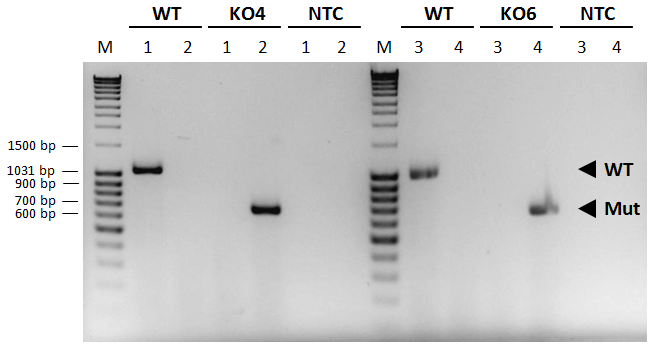 | **(c)**  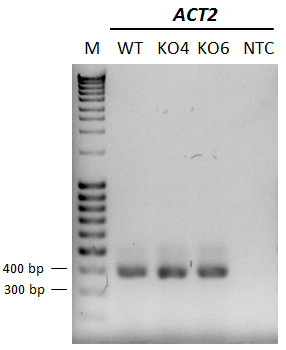 |

**Fig. ST1** Selection of transgenic KO lines. (a), Genomic organization of the *F-box-Nictaba* gene *(At2g02360)* (1-1363 bp) with indicated sites of T-DNA insertions within the gene as in the mutant lines KO4 (SALK_007866) and KO6 (SALK_085735C). Binding positions for primers used for testing of mutant lines are marked with arrows and corresponding primer names. (b), PCR on genomic DNA of WT plants and mutant T-DNA insertion lines KO4 and KO6 demonstrating homozygosity of the transgenic lines. Lanes 1: Primer combination LP_KO4_+RP_KO4_; Lanes 2: Primer combination LBb1.3 + RP_KO4_; Lanes 3: Primer combination LP_KO6_+RP_KO6_; Lanes 4: Primer combination LBb1.3 + RP_KO6._ WT: WT allele, Mut: mutant allele. (c), DNA quality tested with actin (*ACT2*) primers. M: DNA marker; NTC: no template control.

Next, RT-PCR and qRT-PCR were performed to analyze whether the *F-box-Nictaba* transcript is indeed absent in the two presumed KO lines. As presented in Fig. ST2a, the full-length F-box-Nictaba sequence (819 bp) could be amplified both in cDNA of WT plants and of KO4 plants, but not in cDNA samples of KO6 plants. Figure ST2b clearly demonstrates that *F-box-Nictaba* transcript levels in the KO4 line are indeed not reduced but are 3-fold higher than in the WT plants. In contrast to line KO4, *F-box-Nictaba* expression in the KO6 mutant line is over 20-fold lower than in the WT plants. Altogether, these data demonstrate that the KO6 line is a true KO line and that despite T-DNA insertion, *F-box-Nictaba* is still expressed in the KO4 plants. Most probably, the T-DNA sequence present in the *At2g02360* sequence in the KO4 line was not inserted at the predicted insertion point, but is rather introduced at the 3’UTR of the gene where it does not prevent *At2g02360* from transcript expression. Therefore, these KO4 plants were excluded from subsequent plant phenotypic and physiological assays.

**(a) (b)**


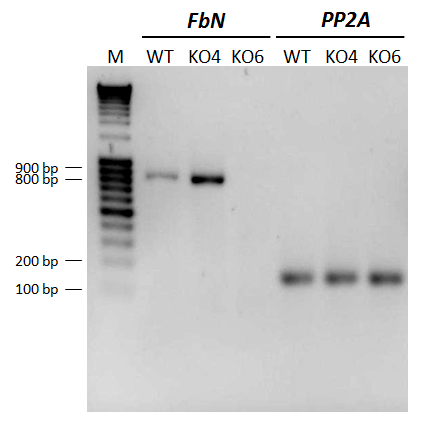


Fig. ST2 Analysis of *F-box-Nictaba* transcripts in putative KO lines. (a), RT-PCR on cDNA isolated from 3-week-old WT plants and from mutant T-DNA insertion lines KO4 and KO6. M: DNA marker; *FbN:* *F-box-Nictaba* gene*; PP2A: protein phosphatase 2* reference gene. (b), Relative expression of *F-box-Nictaba* in 3-week-old *A. thaliana* plants of selected SALK lines designated as KO4 (SALK_007866) and KO6 (SALK_085735C) in comparison to WT plants. Expression analysis was determined by qRT-PCR on a pooled sample of 10 plants; *n=*1; error bars ± SE.

Validation of the F-box-Nictaba-specific antibody

Transgenic lines with enhanced expression of the F-box-Nictaba gene *At2g02360* were created as described in the main text. In order to confirm the over-expression of F-box-Nictaba, a specific antibody was required which could distinguish the F-box-Nictaba protein encoded by *At2g02360* from the other highly homologous F-box proteins with a Nictaba domain [6]. To avoid aspecific reactivity, an antibody against F-box-Nictaba has been developed based on the peptide ^201^cfseairrgrrnvvkpkqre^220^ present in the most distinct region of the Nictaba domain of the F-box-Nictaba sequence.

Out of two selected immunized guinea pigs, the serum of only one of the animals was reactive against the recombinantly expressed *A. thaliana* F-box-Nictaba proteins [4] (Fig. ST3, lanes 1 and 2) without detection of the tobacco lectin Nictaba [5] (Fig. ST3, lane 5) and producing only a moderate background in the lanes containing total protein extracts from WT *A. thaliana* plants and cells (Fig. ST3, lanes 3 and 4). The negative control, where serum before immunization was used as primary antibody, returned no signal at all with little background (Results not shown). The peptide-based antibody should therefore be specifically directed against the F-box-Nictaba protein from *A. thaliana*.

| 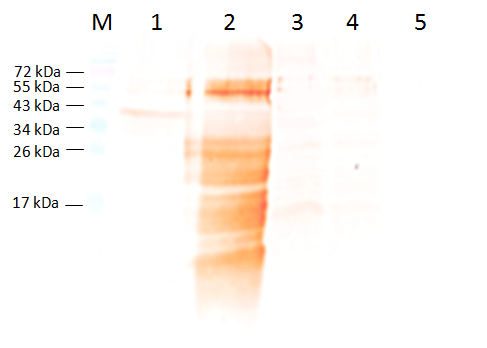 |
| --- |

Fig. ST3 Specificity test of the anti-F-box-Nictaba antibody by Western blot using the serum of immunized animal PY0147 (1/500 dilution) as primary antibody. M: protein marker; Lane 1: purified recombinant F-box-Nictaba (1 µg) [4]; Lane 2: purified recombinant Nictaba domain of F-box-Nictaba (1 µg) [4]; Lane 3: total protein extract from WT *A. thaliana* plants (50 µg); Lane 4: total protein extract from WT *A. thaliana* cells (50 µg); Lane 5: purified recombinant Nictaba protein from tobacco plants (1 µg) [5].

**The *F-box-Nictaba* gene *At2g02360* is highly up-regulated in the generated OE lines**

Five homozygous transgenic *A. thaliana* lines of T4 generation were selected on kanamycin. qRT-PCR analysis was performed to quantify *F-box-Nictaba* gene overexpression. Figure ST4a demonstrates that all five lines overexpress the *F-box-Nictaba* gene. The highest expression is presented by lines OE9 and OE6, reaching almost 300- and 250-fold up-regulation, respectively. Lines OE2, OE4 and OE11 show lower but still very high overexpression of the *F-box-Nictaba* reaching transcript levels of approximately 80-100 times higher than in the WT plants.

Two lines, OE4 and OE6, showing different levels of *F-box-Nictaba* gene overexpression were selected for further phenotypic analyses of transgenic plants. Prior to experiments, both lines were checked by Western blot for the presence of elevated amounts of the F-box-Nictaba protein. As shown in Fig. ST4b, a distinct band of approximately 70 kDa was visible in protein extracts of the OE6 line. No clear and distinct signal was detectable in the protein extract of OE4 plants. Nonetheless, as revealed by qRT-PCR analysis (Fig. ST2a), the overexpression level of *F-box-Nictaba* in line OE4 is approximately 2.5 times lower than that in the OE6 line. Therefore, the Western blot analysis has been repeated with double amount of total protein loaded. Although the background signal increased due to the high protein load, a separate band of 70 kDa was distinguishable in the OE4 lane but not in the protein extracts from both the KO6 and WT plants (Fig. ST4c). Therefore, despite relatively low levels of recombinant protein synthesis, both OE lines overexpress F-box-Nictaba and were considered suitable for further experiments.

| **(a)**   | **(b)**  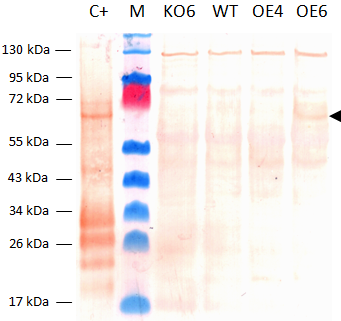 | **(c)**  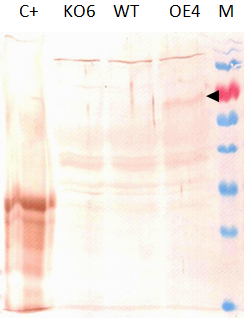 |
| --- | --- | --- |

Fig. ST4 Expression analysis of *F-box-Nictaba* gene and protein in 3-week-old *A. thaliana* OE plants. (a), Relative expression of *F-box-Nictaba* in five different OE lines in comparison to WT plants. Expression analysis was determined by qRT-PCR on a pooled sample of 10 plants; *n=*1; error bars ± SE. (b-c), Western blot of total protein extracts from WT *A. thaliana* plants and transgenic knockout (KO6) and overexpression (OE4 and OE6) plants immunodetected with the anti-F-box-Nictaba antibody. (b), Immunodetection of F-box-Nictaba in 50 µg of total protein extracts from WT, KO6 as well as OE4 and OE6 plants. (c), Immunodetection of F-box-Nictaba in 100 µg of total protein extracts from WT, KO6 and OE4 plants. M: protein marker; C+: positive control - purified Nictaba domain of F-box-Nictaba (1 µg). The position of the polypeptide presumably corresponding to F-box-Nictaba is indicated with black marker.

####

#### Transgenic plants perform similar as WT plants during normal growth conditions

Neither the KO6 line nor the two over-expression lines showed an obvious altered phenotype throughout development of plants grown under optimal conditions. *In vitro* germination assays did not reveal any differences in transgenic seed germination rates in comparison to the seeds of WT *A. thaliana* plants (Fig. ST5a-b). Similarly, measurements of the size of fully developed rosette leaves showed that altered *F-box-Nictaba* gene expression did not affect vegetative development of *A. thaliana* plants (Fig. ST5c-d).

**Fig. ST5** Phenotypic analyses of transgenic plants with altered *F-box-Nictaba* expression. (a) and (b), Assessment of seed germination rates performed by daily calculation of the percentage of germinated seeds starting from the first day of incubation in the growth chamber until day 5. The analysis included two independent biological replicates. *n*=2; error bars ± SE. C and D, Measurement of rosette leaf size in 5-week-old plants. The analysis included two independent biological replicates. *n*=2; error bars ± SE. Statistical analyses were performed using the Student’s t-test (p<0.05).

**References**

**[1]** Alonso JM, Stepanova AN, Leisse TJ, Kim CJ, Chen H, Shinn P, Stevenson DK, Zimmerman J, Barajas P, Cheuk R, Gadrinab C, Heller C, Jeske A, Koesema E, Meyers CC, Parker H, Prednis L, Ansari Y, Choy N, Deen H, Geralt M, Hazari N, Hom E, Karnes M, Mulholland C, Ndubaku R, Schmidt I, Guzman P, Aguilar-Henonin L, Schmid M, Weigel D, Carter DE, Marchand T, Risseeuw E, Brogden D, Zeko A, Crosby WL, Berry CC, Ecker J.R. Genome-wide insertional mutagenesis of *Arabidopsis thaliana*. Science 2006;301:653-657.

**[2]** Van Hove J, De Jaeger G, De Winne N, Guisez Y, Van Damme EJM The Arabidopsis lectin EULS3 is involved in stomatal closure. Plant Sci. 2015;238:312-322.

**[3]** Van Hove J, Stefanowicz K, De Schutter K, Eggermont L, Lannoo N, Al Atalah B, Van Damme EJM. Transcriptional profiling of the lectin ArathEULS3 from *Arabidopsis thaliana* towards abiotic stresses. J Plant Physiol. 2014;171:1763-1773.

**[4]** Stefanowicz K, Lannoo N, Proost P, Van Damme EJM. Arabidopsis F-box protein containing a Nictaba-related lectin domain interacts with *N-*acetyllactosamine structures. FEBS Open Bio 2012;2:151-158.

**[5]** Schouppe D, Rougé P, Lasanajak Y, Barre A, Smith DF, Proost P, Van Damme EJM. Mutational analysis of the carbohydrate binding activity of the tobacco lectin. Glycoconj J. 2010;27:613-623.

**[6] Delporte A, Van Holle S, Lannoo N, Van Damme EJM. The tobacco lectin, prototype of the family of Nictaba-related proteins. Curr Prot Pept Sci. 2015;16:5-16.**
